# Life expectancy tables for dogs and cats derived from clinical data

**DOI:** 10.3389/fvets.2023.1082102

**Published:** 2023-02-21

**Authors:** Mathieu Montoya, Jo Ann Morrison, Florent Arrignon, Nate Spofford, Hélène Charles, Marie-Anne Hours, Vincent Biourge

**Affiliations:** ^1^Royal Canin, Aimargues, France; ^2^Banfield Pet Hospital, Vancouver, WA, United States; ^3^MAD Environnement, Nailloux, France

**Keywords:** mortality, survival, lifespan, obesity, longevity

## Abstract

There are few recent and methodologically robust life expectancy (LE) tables for dogs or cats. This study aimed to generate LE tables for these species with clinical records from >1,000 Banfield Pet hospitals in the USA. Using Sullivan's method, LE tables were generated across survey years 2013–2019, by survey year, and for subpopulations defined by sex, adult body size group (purebred dogs only: toy, small, medium, large and giant), and median body condition score (BCS) over life. The deceased population for each survey year comprised animals with a recorded date of death in that year; survivors had no death date in that year and were confirmed living by a veterinary visit in a subsequent year. The dataset totaled 13,292,929 unique dogs and 2,390,078 unique cats. LE at birth (LE_birth_) was 12.69 years (95% CI: 12.68–12.70) for all dogs, 12.71 years (12.67–12.76) for mixed-breed dogs, 11.18 years (11.16–11.20) for cats, and 11.12 (11.09–11.14) for mixed-breed cats. LE_birth_ increased with decreasing dog size group and increasing survey year 2013 to 2018 for all dog size groups and cats. Female dogs and cats had significantly higher LE_birth_ than males: 12.76 years (12.75–12.77) vs. 12.63 years (12.62–12.64), and 11.68 years (11.65–11.71) vs. 10.72 years (10.68–10.75), respectively. Obese dogs (BCS 5/5) had a significantly lower LE_birth_ [11.71 years (11.66–11.77)] than overweight dogs (BCS 4/5) [13.14 years (13.12–13.16)] and dogs with ideal BCS 3/5 [13.18 years (13.16–13.19)]. The LE_birth_ of cats with BCS 4/5 [13.67 years (13.62–13.71)] was significantly higher than cats with BCS 5/5 [12.56 years (12.45–12.66)] or BCS 3/5 [12.18 years (12.14–12.21)]. These LE tables provide valuable information for veterinarians and pet owners and a foundation for research hypotheses, as well as being a stepping-stone to disease-associated LE tables.

## 1. Introduction

Human health metrics and life expectancy computations have been used for centuries. The first quantification of life expectancy was reported in England in the 19th century (1). Life expectancy tables have diverse uses for humans, which include monitoring the overall health status of populations over time, exploring health inequalities in socio-economic groups, and charting the progress of less developed nations (2, 3). They are valuable for understanding the acute and long-term, direct and indirect effects of health conditions and medical interventions on survival. Life expectancy data can be used to inform planning for healthcare provisions, economic policies, life insurance premiums and pension annuities.

Accurately calculating the life expectancy of dogs and cats would be of greatest interest in helping to understand the benefits of preventative care and the success of increasingly advanced medical interventions. However, there are relatively few studies on lifespan or all-cause mortality in either of these species, especially in cats. Four studies in dogs have compiled age of death statistics from surveys of owners belonging to the UK Kennel club (4, 5), the Danish Kennel club (6) and owners on a UK insurance database or attending the Crufts dog show (7). Analyses of the primary-care veterinary records of both dogs (8) and cats (9) in England have provided age of death data as the percentage of pets dying within pre-specified age brackets. Kaplan-Meier survival curves for dogs attending the Banfield Pet Hospital network of primary care hospitals in the USA have been published (10), and a study of life-insured Swedish cats generated survival curves for cats using a methodology of age standardized cox regression (11). These studies have provided valuable data, but they do not provide the same information as life expectancy tables, which are a standard way of gauging the health status of large human populations.

Survival data are analyzed in two ways: the life-table method divides time into intervals and calculates survival in each interval; the Kaplan-Meier method calculates survival each time an event occurs (death). Both methods produce a graph (survival curve) that shows the cumulative probability (hazard ratio) of the event in the total period of observation. Kaplan-Meier survival analysis may be more appropriate for studies with longitudinal follow-up focused on the time until events occur. In contrast, the life-table methodology is a statistical technique that provides a summary of the mortality and life expectancy experiences of a large population of different ages. This study used life table methodology to analyze survival, in part to provide information equivalent to that for large human populations, and in part because of the high dropout rates of animals (right-censored individuals relative to animals with a confirmed date of death) due to discontinuity in veterinary visits in the clinical dataset.

Life expectancy tables for pets have been reported previously from a total of four studies in Japan and one in the UK (12–16). The oldest of these studies generated a life expectancy table using the cemetery records of 4,915 dogs in the Kyoto region of Japan that died between 1981 and 1982 (12). Similarly there was a census of 12,039 dogs from cemeteries in Tokyo between 2012 and 2015 (14). The only life expectancy tables found for cats were also from Japanese cemetery data in Tokyo (3,936 cats identified from between 1981 and 1982) (16). Using such data sources comprising only dead animals for a life-table methodology is unsatisfactory because of a “hidden” bias of right censorship, since they cannot account for animals in a cohort still alive at the time of sampling (17). This limitation did not apply to the fourth Japanese study, which generated life expectancy tables from the insurance records of 299,555 dogs insured between 2010 and 2011 and followed for 1 year (13). The first life tables for dogs in the UK were based on the VetCompass™ database of primary-care veterinary clinics, in which there were 30,563 dogs with at least one clinical record in 2016 and a confirmed death between January 2016 and July 2020 (15). Despite differences between studies in data sources and the computed life expectancy, they all found substantial differences in life expectancies between different dog sizes or breeds when this was investigated.

Other studies in dog populations comprising multiple breeds and both sexes have summarized overall ages of death without computing life expectancy tables (4–8). Age of death was 10 years (median) in a Danish survey of 2,928 purebred and mixed-breed dogs in 1997 (6), 10.33 years (median) in a survey of 5,663 Kennel Club-registered dogs in the UK in 2014 (4), 11.08 years (mean) in 3,000 British purebred and mixed-breed dogs (7), 11.25 years (median) in 15,881 purebred dogs in the UK over the 10 year prior to 2003 (5), and 12 years (median) in a UK VetCompass™ dataset of 5,095 dogs between 2009 and 2011 (8). Age-at-death data are scarce for cat populations comprising multiple breeds and/or mixed-breed cats. A study that used a random sample of 4,009 purebred and mixed-breed cats with confirmed deaths in the UK VetCompass™ database between 2009 and 2012 reported a median age of death of 14.0 years [interquartile range (IQR): 9.1–17.0] (9).

The more recent studies demonstrate the opportunities available in the current era of veterinary “big data”, which is giving researchers access to reliable and extensive high quality data over long periods of time (18). There is a need now to leverage the power of other large datasets to characterize life expectancy in countries with different disease risks, attitudes toward veterinary care, and accessibility of such care. Furthermore, life expectancy tables are needed for pets with and without specific known or hypothesized risk factors for disease, to help quantify the potential benefits of improving modifiable risk factors, and to support conversations with owners on proactive lifestyle choices for the health of their pets. Obesity in pets is a condition of particular interest. It is the most common nutritional disorder in companion animals (19), and obese dogs have a reduced lifespan compared with dogs of normal body condition (20), while severe obesity in cats is associated with reduced survival and reduced lifespan (21). Obesity in pets is associated with a wide range of functional impairments and health conditions (22–30) that could potentially have a direct or indirect impact on life expectancy. Examples include associations with diabetes mellitus (25, 26), metabolic derangements similar to some of those seen in metabolic syndrome in humans (31), and changes in the structure and function of the heart (29) and kidneys (24). The cause and effect of associations between obesity, health and reduced life expectancy or lifespan is a multifaceted and complex field of active research in both humans and pets. Understanding the impact of obesity as either a risk factor for the development of specific diseases or as a consequence of them is important for preventative medicine, disease signalment and disease management.

The current study aimed to generate life expectancy tables for dogs and cats in the USA, computed for each survey year between 2013 and 2019, and across survey years according to breed size, sex, and body condition score (BCS). Clinical data were obtained from the electronic medical records (EMRs) of healthy and sick dogs and cats attending Banfield hospitals throughout the USA. Computations followed Sullivan's method for life expectancy tables, which can also account for health status assessed as a cross-sectional variable (32). This methodology is one of the most popular methods for life expectancy tables and is used by the World Health Organization.

## 2. Materials and methods

### 2.1. Study populations

The clinical dataset was derived from the EMRs of 1,152 different Banfield hospitals in the USA between January 1st, 2013, and July 31st, 2022. The number of hospitals varied between years due to the closure of some hospitals and the opening of new ones, however most hospitals contributed data for each of the 9 years of the study (*n*_2013_ = 854; *n*_2014_ = 893; *n*_2015_ = 933; *n*_2016_ = 984; *n*_2017_ = 1,015; *n*_2018_ = 1,044; *n*_2019_ = 1,074; *n*_2020_ = 1,084; *n*_2021_ = 1,069; *n*_2022_ = 1,054). All hospitals were linked by a proprietary practice management system (PetWare^®^) and data from all visits were uploaded to a central database, resulting in a large amount of aggregated, structured data.

The dataset was cleaned by removing animals with no recorded birth date, anomalous birth or death dates (date of birth posterior to visit, or date of death anterior to visit), a visit age >30 years, or incomplete data for sex, age or BCS. The resulting dataset is referred to as the cleaned dataset.

**Figure 1:**
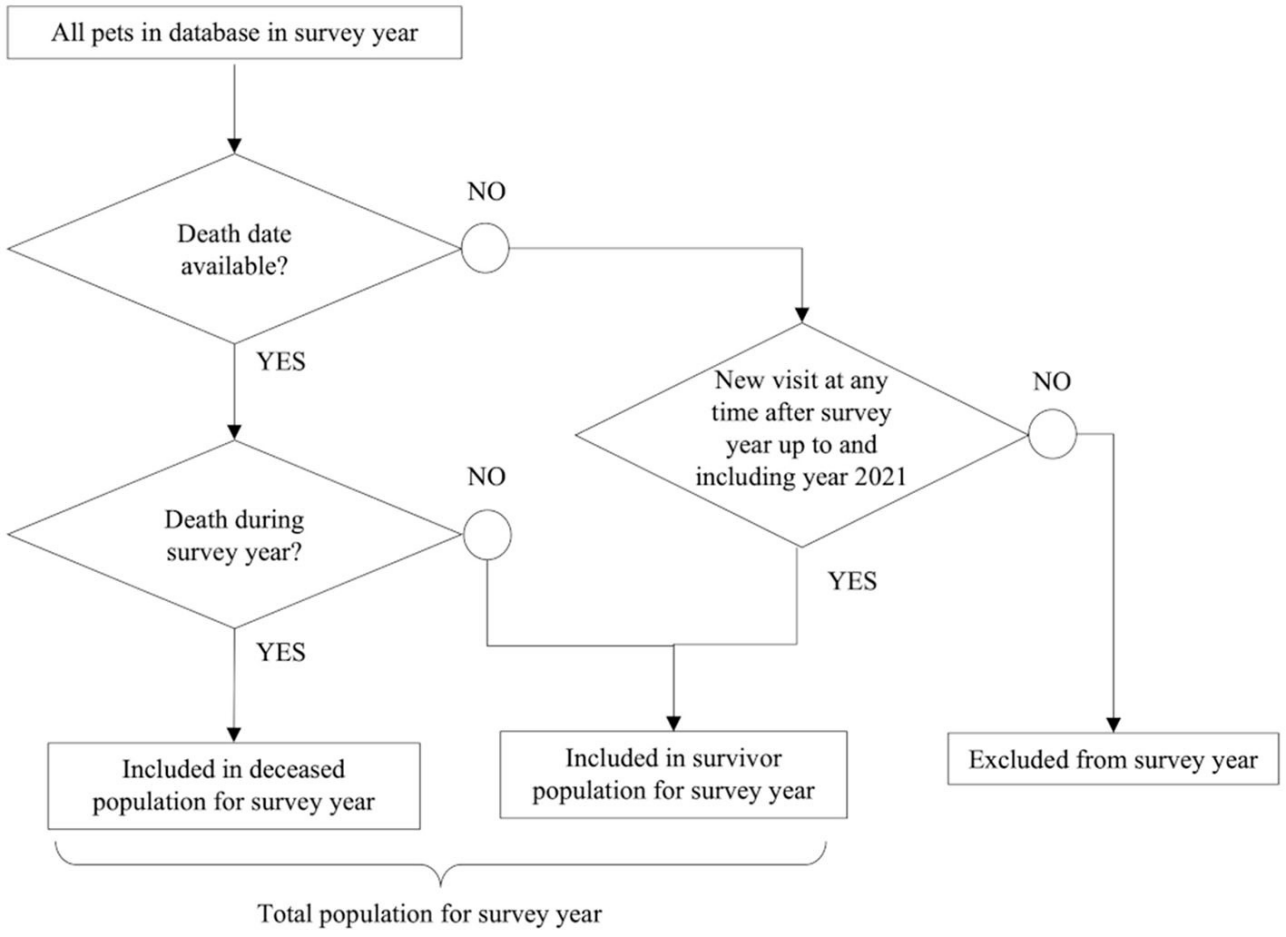
Methodology used to build the study population in each calendar survey year from the clinical dataset.

A deceased and survivor population for each survey year was identified from the clinical dataset as outlined in Figure 1; the sum of these two populations comprised the total study population for that year. Dogs and cats with no death date in the EMRs were only considered to be survivors for a specific year if there was a new hospital visit in any of the following study years. Dogs and cats with no death date and that only visited the clinic in the course of 1 year, with no return visits in a subsequent year of the study, were excluded because they could not be confirmed as either a survivor in any year or a deceased animal.

The return rate of animal visits across all study years was calculated to support the validity of the range of years selected for the analysis. On average, dogs returned in 111 days and cats in 146 days; median return rate was 70 days (IQR: 21–169) for dogs and 90 days (21–190) for cats. The Tukey's outlier detection method applied to the return rate in days yielded an upper fence (*UF* = *Q*3 + 1.5^*^IQR) of 391 days for dogs and 443 days for cats. At least two full years of data after a survey year were needed for life table calculation. It was decided to analyze life expectancy in the dataset from January 1st, 2013 to December 31st, 2019. The last 2.5 years of data (January 1st, 2020 to July 31st, 2022) were used to define if a pet without a death date could be confirmed as a survivor in any of the previous years.

Subpopulations of each species were defined by sex (male or female), and median BCS was recorded for individuals between January 1st, 2013 and July 31st, 2022. The sterilization status was available in the clinical dataset, but it was not included in the study because of the way these data were collected. Pets that were sterilized at a certain age were identified as neutered from birth to death. This introduced a bias due to mortality in the first few months of life when pets were not yet spayed or neutered, which impacted the early mortality rate for entire animals. Body condition score was assessed on a 5-point scale between 2013 and 2018, and on a 9-point scale between 2018 and 2022. Data from the 9-point scale were converted to the 5-point scale (Table 1). Separate analyses were conducted for purebred and mixed-breed animals. Mixed-breed dogs were recorded as such in the database, whereas cats were identified as mixed breed if they were recorded as Domestic Short Hair, Domestic Long Hair, or Domestic Medium Hair.

**Table 1:** Conversion of BCS 9-point scale to BCS 5-point scale.

**BCS 9-point scale**		**BCS 5-point scale**	
**Score**	**Description**	**Score**	**Description**
1	Emaciated	1	Very thin
2	Very thin	2	Underweight
3	Thin		
4	Ideal weight	3	Ideal weight
5	Ideal weight		
6	Overweight	4	Overweight
7	Heavy		
8	Obese	5	Obese
9	Severely obese		

BCS, body condition score.

In addition, the population of purebred dogs was subdivided into five size categories based on adult bodyweight: toy, small, medium, large and giant. Firstly, dogs weighing more than 70 and <0.5 kg were removed from the uncleaned dataset of purebred dogs. Bodyweight outliers for each breed were then excluded using the Tukey's outlier detection method (lower fence = *Q*1 − 1.5^*^IQR; upper fence = *Q*3 + 1.5^*^IQR). The average adult bodyweight of each breed in the resulting dataset was calculated from dogs aged 2–5 years, and was used to assign a size group (Table 2) to each breed in the cleaned dataset.

**Table 2:** Dog size groups.

**Size group**	Adult bodyweight^*^	**Number of breeds in size group**
Toy	< 5.5 kg	19
Small	≥5.5 to < 11 kg	94
Medium	≥11 to < 26 kg	215
Large	≥26 to < 45 kg	154
Giant	≥45 kg	26
Mixed breed	All adult bodyweights	1 (mixed breed)

* Adult bodyweight was taken to be the bodyweight at 2–5 years of age.

### 2.2. Construction of life tables

Life expectancy tables for dogs and cats were generated using the probabilistic approach of Sullivan's method (32), based on the deceased and survivor populations of dogs and cats described in Section 2.1. This methodology is applied in studies on human health assessments (33–38). Uncertainties were assessed with Monte Carlo methods (33, 34, 37, 39, 40).

The variables used in life table calculations were the number of dead pets *d*(*x*) in a 1-year age interval (*x, x*+1), and the study population size (dead plus living pets) at the mid-year point *P*(*x*). Central death rate was computed as $m\left(x\right)=\;\frac{d\left(x\right)}{P\left(x\right)}$. Pets who died in the interval (*x, x*+1) had lived *x* complete years plus a fraction *a*(*x*) of the last year. It was assumed that deaths were uniformly distributed in each year interval, occurring on average midway in the age range (*x, x*+1); therefore *a*(*x*) was uniformly taken to be 0.5 (41).

The conditional probability of death in a 1-year age interval (*x, x*+1) was calculated as $q\left(x\right)=\;\frac{m\left(x\right)}{\left(1+a{\left(x\right)}^{*}m\left(x\right)\right)}$. The number of pets surviving to age *x*, denoted as *l*(*x*), was estimated from a hypothetical population of 100,000 pets at birth i.e., *l*(0) = 100, 000. As these pets were assumed to be subject throughout their lives to the conditional probability of death in the 1-year age interval (*x, x*+1), the calculation was *l*(*x*) = (1−*q*(*x*−1))^*^l(*x*−1). The number of pet years lived at age *x* (any age other than the first year of life and the final age interval), denoted as *L*(*x*), was the sum of pets surviving until age (*x*+1) and the fraction *a*(*x*) of pets surviving in age interval (*x, x*+1), so that *L*(*x*) = *l*(*x*+1)+*a*(*x*)^*^(*l*(*x*)−*l*(*x*+1)). Given that *a*(*x*) = 0.5, the equation was simplified to $L\left(x\right)=\frac{\left(l\left(x\right)+l\left(x+1\right)\right)}{2}$. For the first year of life, the assumption was made that 80% of the deaths happened in the first months of life, leading to *L*(0) = 0.2^*^l(0)+0.8^*^l(1). For the last age interval ω, as no information was available after this age, it was calculated as $L\left(\omega \right)=\frac{l\left(\omega \right)}{m\left(\omega \right)}$. The total number of years lived beyond age *x* was $T\left(x\right)=\;\overset{\omega }{\underset{i=x}{\sum }}L\left(x\right)$. The final equation for life expectancy at year *x* was $e\left(x\right)=\frac{T\left(x\right)}{l\left(x\right)}$. Life expectancy at birth (LE_birth_) corresponded to life expectancy for animals in the 0–1 age interval. Uncertainties were calculated with Monte-Carlo methods, with *n* = 1.10^6^ iterations, drawing *d*(*x*) from a parametrized binomial distribution where each sample was equal to the number of successes over *n* trials, the probability of success being $\frac{d\left(x\right)}{P\left(x\right)}$ and the number of successes *P*(*x*). Life expectancy *e*(*x*) was calculated *n* times, using the Monte-Carlo-derived *d*(*x*) in the previous equations. Confidence intervals (CIs) at the 95% level were calculated for each year using the mean *e*(*x*) and standard deviation [denoted as *S*_*LE*_(*x*)] for the *n* iterations: $CI_{95\%}\left(x\right)=e\left(x\right)\pm \;1.96^{*}S_{LE}\left(x\right)$.

Life tables were generated for each species across all survey years, for each survey year, for the global population and the subpopulations defined in Section 2.1. For each subpopulation, the variables *P*(*x*) and *d*(*x*) specific to that subpopulation were used to recalculate the intermediate variables, *m*(*x*), *q*(*x*), *l*(*x*), *L*(*x*) and *T*(*x*). Only data for purebred dogs were used for life expectancy estimations by dog size group.

### 2.3. Software applications

Only structured data were extracted from the PetWare^®^ database; there was no searching of free text entries. Software applications used for data processing and building life tables were Databricks (runtime 11.0, with Apache Spark 3.3.0) on Microsoft Azure, with Python 3.9.5 (libraries Numpy 1.20.3, Pandas 1.3.4, Lifelines 0.27.2 and Plotly 5.6.0).

## 3. Results

### 3.1. Study population

**Figure 2:**
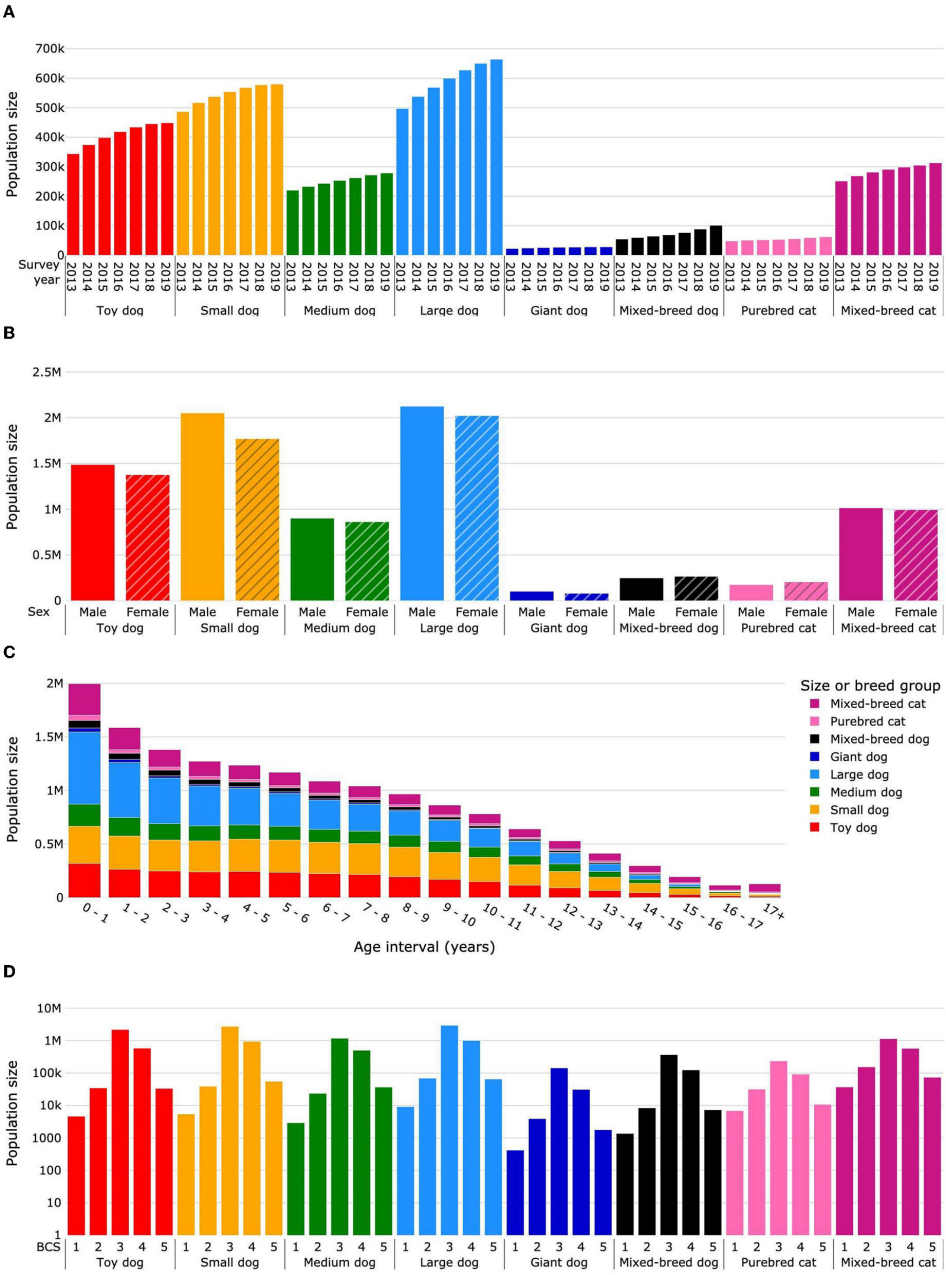
Total population sizes (deceased and survivor) for life expectancy calculations. Dogs were categorized as purebred dogs in size groups toy, small, medium, large and giant, and as mixed-breed dogs. Cats were categorized as purebred and mixed-breed cats. Population sizes are presented by survey year **(A)**, sex **(B)**, age interval **(C)**, and median body condition score **(D)**. Body condition scoring was on a 5-point scale from 1 = very thin to 5 = obese. BCS, body condition score.

There were 64.76 million PetWare^®^ visit records for 9.29 million unique dogs, and 9.85 million visits for 2.51 million unique cats. Of these animals, 1.74% dogs and 1.82% cats were removed to produce the cleaned dataset as described in the methods. The total population sizes (deceased and survivor combined) in the cleaned dataset are presented by survey year for each species in Figure 2A and Supplementary Table 1. Pets had a mean 2.5 visits during a survey year, and over the 9 years studied had a mean 6.3 visits and a median of three visits (IQR: 1–8). The percentage of dogs returning within 2 years after the survey year increased from 74.8% in 2013 to 77.6% in 2017, and subsequently decreased to 75.9% in 2019. For cats, the percentage of returns was 61.9% in 2013, it increased each year to a maximum of 65.4% in 2017, then it dropped to 62.5% in 2019 (Supplementary Table 2).

In summary, the mean total dog population size (deceased and survivor populations) was 1,899,048 individual dogs per year (26,099 giant, 592,413 large, 251,831 medium, 545,988 small, 409,204 toy, and 73,513 mixed breed). The mean deceased dog population was 85,651 per year, and a mean of 621,284 (25%) dogs were excluded each year on account of having no date of death and no additional visits in subsequent years (Figure 1). The mean age at death for dogs was 10.66 years, the median age at death was 11.57 years (IQR: 8.4–13.9), and the median survival time was 14.13 years.

The mean total population size for cats was 314,823 individuals per year (54,478 purebred and 287,345 mixed breed). The mean deceased cat population was 28,308 cats per year, and 172,523 (34%) cats per year were excluded according to the study methodology (Figure 1). The mean age at death was 11.15 years, the median age at death was 12.30 years (IQR: 7.0–15.7), and the median survival time was 15.7 years.

The overall proportion of male dogs was 52.0%; for purebred dogs it was 51.9, 53.7, 51.1, 51.2, and 55.9% for toy, small, medium, large and giant sizes, respectively, and for mixed-breed dogs it was 48.3% (Figure 2B; Supplementary Table 3). The proportion of males in the total cat population was 49.8% (45.9% for purebred and 50.5% for mixed-breed cats). In total, 91.3% of female and 86.6% of male dogs were sterilized. The percentage of sterilized cats was 96.1% for females and 96.9% for males.

Data were sparse for dogs and cats older than 17 years, and uncertainty in life expectancy calculations became unacceptably large. Therefore, dogs and cats living more than 17 years were grouped into an age interval of 17+ years. Figure 2C and Supplementary Table 4 show the distribution of population headcount by pet group and age interval. The cat population was smaller in each age interval compared with the dog population except for the age interval 17+ years, in which the cat population was more than two times larger than the dog population.

The median BCS was 3 for 72.7% of dogs (toy 77.0%, small 72.4%, medium 67.6%, large 71.9%, giant 79.4%, and mixed breed 72.3%) (Figure 2D; Supplementary Table 5). The prevalence of obesity (median BCS 5) was 1.5% (toy 1.2%, small 1.5%, medium 2.1%, large 1.6%, giant 1.0%, and mixed breed 1.4%). In the cat population, the median BCS was 3 for 58.5% of animals (purebred 62.4%, 57.7% mixed breed) (Figure 2D; Supplementary Table 5) and the prevalence of obesity (median BCS 5) was 3.6% (2.9% purebred, 3.7% mixed breed). The percentage of dogs with median BCS 3 increased each year from 68.7% in 2013 to 74.5% in 2019, while for median BCS 5 it decreased from 2.1% in 2013 to 1.3% in 2017, subsequently remaining constant until 2019. The percentage of cats with median BCS 3 increased from 53.2 to 60.3% between 2013 and 2019, whereas for median BCS 5 it decreased from 4.3% in 2013 to 3.5% in 2015, and then fluctuated between 3.5 and 3.3% until 2019 (Supplementary Table 6).

In summary, the study dataset for dogs covered all bodyweight size categories; the large and small dog sizes had the greatest representation. Less than 5% of dogs were of mixed breed, whereas in the cat dataset there were ~5 times more mixed-breed cats than purebred. For both cats and dogs the sex ratio was well-balanced between males and females, and the vast majority of both sexes in both species were sterilized. Median body condition was ideal for nearly three quarters of dogs and obese for 1.5%. In contrast, for cats, median body condition was ideal for slightly more than two thirds and obese for 3.6%.

### 3.2. Life expectancy tables

The global life expectancy table with intermediate variables is presented in Table 3 for dogs and Table 4 for cats. The life expectancy of dogs decreased by ~0.9 years each age interval between 1–2 and 7–8 years. Subsequently the decrease in life expectancy diminished from interval to interval, and from 12 to 13 years onwards, the decrease was <0.5 years per age interval. For cats, life expectancy decreased by approximately 0.7 years each age interval between 1–2 and 8–9 years. After this, the reduction in life expectancy decreased, and was 0.5 years or less after the 10–11 years age interval.

**Table 3:** Global life expectancy table for dogs (all purebred and mixed breed combined).

**Age interval in years (*x*, *x* + 1)**	***P*(*x*) total population**	***d*(*x*) decease population**	***m*(*x*)**	***q*(*x*)**	***l*(*x*)**	***L*(*x*)**	***T*(*x*)**	***e*(*x*)**	**95% CI**
0–1	1,608,758	43,476	0.0270	0.0267	100,000	97,867	1,269,213	12.69	12.68–12.70
1–2	1,331,906	14,276	0.0107	0.0107	97,334	96,815	1,171,346	12.03	12.03–12.04
2–3	1,177,583	11,310	0.0096	0.0096	96,296	95,836	1,074,532	11.16	11.15–11.17
3–4	1,093,330	11,480	0.0105	0.0104	95,375	94,877	978,696	10.26	10.25–10.27
4–5	1,066,987	13,148	0.0123	0.0122	94,379	93,801	883,819	9.36	9.36–9.37
5–6	1,008,031	16,145	0.0160	0.0159	93,223	92,483	790,017	8.47	8.47–8.48
6–7	935,213	19,637	0.0210	0.0208	91,742	90,789	697,535	7.60	7.60–7.61
7–8	887,776	26,388	0.0297	0.0293	89,836	88,520	606,746	6.75	6.75–6.76
8–9	810,013	35,663	0.0440	0.0431	87,205	85,326	518,225	5.94	5.93–5.95
9–10	707,952	45,257	0.0639	0.0619	83,448	80,863	432,899	5.19	5.18–5.20
10–11	614,524	56,118	0.0913	0.0873	78,279	74,860	352,036	4.50	4.49–4.50
11–12	484,797	60,550	0.1249	0.1176	71,442	67,243	277,175	3.88	3.87–3.89
12–13	371,564	64,343	0.1732	0.1594	63,044	58,020	209,932	3.33	3.32–3.34
13–14	265,424	61,806	0.2329	0.2086	52,997	47,470	151,912	2.87	2.86–2.88
14–15	168,706	51,430	0.3048	0.2645	41,943	36,395	104,442	2.49	2.48–2.50
15–16	92,503	35,442	0.3831	0.3215	30,848	25,888	68,047	2.21	2.19–2.22
16–17	42,472	19,224	0.4526	0.3691	20,929	17,066	42,159	2.01	2.00–2.03
17+	26,010	13,687	0.5262	0.4166	13,204	25,092	25,092	1.90	1.88–1.92

*P*(*x*), total population (survivors plus deceased) at the mid-point of the age interval (*x, x* + 1); *d*(*x*), deceased study population in the age interval (*x, x* + 1); *m*(*x*), central rate of death in the age interval (*x, x* + 1); *q*(*x*), conditional probability of death in the age interval (*x, x* + 1); *l*(*x*), number of a hypothetical population of dogs surviving to the age of *x* years, calculated from a starting population of 100,000 dogs at birth subject throughout life to the same conditional probability of death as the actual population at each age interval; *L*(*x*), number of dog years lived at age *x*, based on the hypothetical starting population of 100,000 dogs at birth; *T*(*x*), number of dog-years lived beyond year *x*; *e*(*x*), life expectancy for dogs in the age interval (*x, x* + 1); CI, confidence interval.

**Table 4:** Global life expectancy table for cats (all purebred and mixed breed combined).

**Age interval in years (*x*, *x* + 1)**	***P*(*x*) total population**	***d*(*x*) decease population**	***m*(*x*)**	***q*(*x*)**	***l*(*x*)**	***L*(*x*)**	***T*(*x*)**	***e*(*x*)**	**95% CI**
0–1	325,096	17,200	0.0529	0.0515	100,000	95,876	1,118,000	11.18	11.16–11.20
1–2	231,549	7,321	0.0316	0.0311	94,846	93,370	1,022,123	10.78	10.75–10.80
2–3	185,553	5,467	0.0295	0.0290	91,893	90,559	928,754	10.11	10.09–10.13
3–4	162,631	4,877	0.0300	0.0295	89,225	87,907	838,194	9.39	9.37–9.42
4–5	150,407	4,827	0.0321	0.0316	86,589	85,222	750,287	8.66	8.64–8.69
5–6	138,833	5,423	0.0391	0.0383	83,854	82,248	665,066	7.93	7.91–7.95
6–7	125,243	5,401	0.0431	0.0422	80,641	78,939	582,818	7.23	7.21–7.25
7–8	118,952	6,291	0.0529	0.0515	77,237	75,247	503,878	6.52	6.51–6.54
8–9	112,659	7,735	0.0687	0.0664	73,258	70,826	428,631	5.85	5.83–5.87
9–10	100,971	8,041	0.0796	0.0766	68,395	65,776	357,805	5.23	5.22–5.25
10–11	99,722	11,352	0.1138	0.1077	63,157	59,755	292,029	4.62	4.61–4.64
11–12	84,860	10,704	0.1261	0.1187	56,354	53,011	232,277	4.12	4.11–4.14
12–13	79,909	13,446	0.1683	0.1552	49,668	45,813	179,263	3.61	3.60–3.62
13–14	70,670	14,571	0.2062	0.1869	41,959	38,037	133,449	3.18	3.17–3.19
14–15	60,980	15,636	0.2564	0.2273	34,116	30,239	95,412	2.80	2.78–2.81
15–16	50,674	15,945	0.3147	0.2719	26,362	22,779	65,173	2.47	2.46–2.49
16–17	37,426	13,940	0.3725	0.3140	19,195	16,181	42,394	2.21	2.19–2.22
17+	57,088	28,678	0.5023	0.4015	13,168	26,213	26,213	1.99	1.97–2.01

*P*(*x*), total population (survivors plus deceased) at the mid-point of the age interval (*x, x* + 1); *d*(*x*), deceased study population in the age interval (*x, x* + 1); *m*(*x*), central rate of death in the age interval (*x, x* + 1); *q*(*x*), conditional probability of death in the age interval (*x, x* + 1); *l*(*x*), number of a hypothetical population of dogs surviving to the age of *x* years, calculated from a starting population of 100,000 dogs at birth subject throughout life to the same conditional probability of death as the actual population at each age interval; *L*(*x*), number of dog years lived at age *x*, based on the hypothetical starting population of 100,000 dogs at birth; *T*(*x*), number of dog-years lived beyond year *x*; *e*(*x*), life expectancy for dogs in the age interval (*x, x* + 1); CI, confidence interval.

### 3.3. Life expectancies by survey year

The LE_birth_ of dogs across all survey years and dog sizes was 12.69 years (95% CI: 12.68–12.70) (Table 2). Life expectancy at birth varied by dog size, being 9.51 years (9.45–9.58) for giant, 11.51 years (11.49–11.52) for large, 12.7 years (12.68–12.72) for medium, 13.53 years (13.52–13.55) for small, and 13.36 years (13.33–13.38) for toy (Supplementary Table 7). Mixed-breed dogs had an LE_birth_ of 12.71 years (12.67–12.76) (Supplementary Table 7). The LE_birth_ of cats across all survey years was 11.18 years (11.16–11.20) (Table 2). Life expectancy at birth varied by cat group, being 11.54 years (11.48–11.6) for purebred and 11.12 years (11.09–11.14) for mixed-breed cats (Supplementary Table 7).

**Figure 3:**
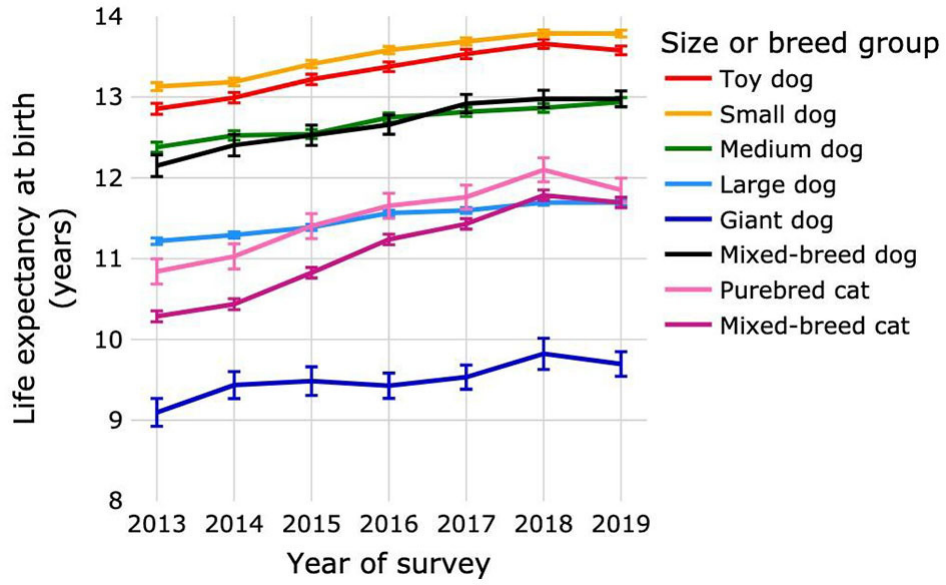
Life expectancies at birth of purebred and mixed-breed dogs and cats by survey year. Dogs were categorized as purebred dogs in size groups toy, small, medium, large and giant, and as mixed-breed dogs. Cats were categorized as purebred and mixed-breed cats. Error bars represent 95% confidence intervals.

The LE_birth_ increased progressively for survey years 2013–2018 for all dog size groups and for cats (Figure 3; Supplementary Table 7). A small decrease in life expectancy was observed in 2019 for toy and giant dog size groups and also for cats; the decreases were not significant for either species. Changes in cat LE_birth_ over successive survey years were greater than those for all size groups of dogs in all time periods, with the exception of the change between 2013 and 2014 for giant dogs. Across the entire study period, LE_birth_ increased by 0.72 years (5.62%), 0.66 years (5.0%), 0.56 years (4.51%), 0.48 years (4.27%), 0.6 years (6.59%), and 0.83 years (6.81%) for toy, small, medium, large, giant and mixed-breed dogs, respectively, and by 1.01 years (9.31%) for purebred and 1.41 years (13.69%) for mixed-breed cats.

**Figure 4:**
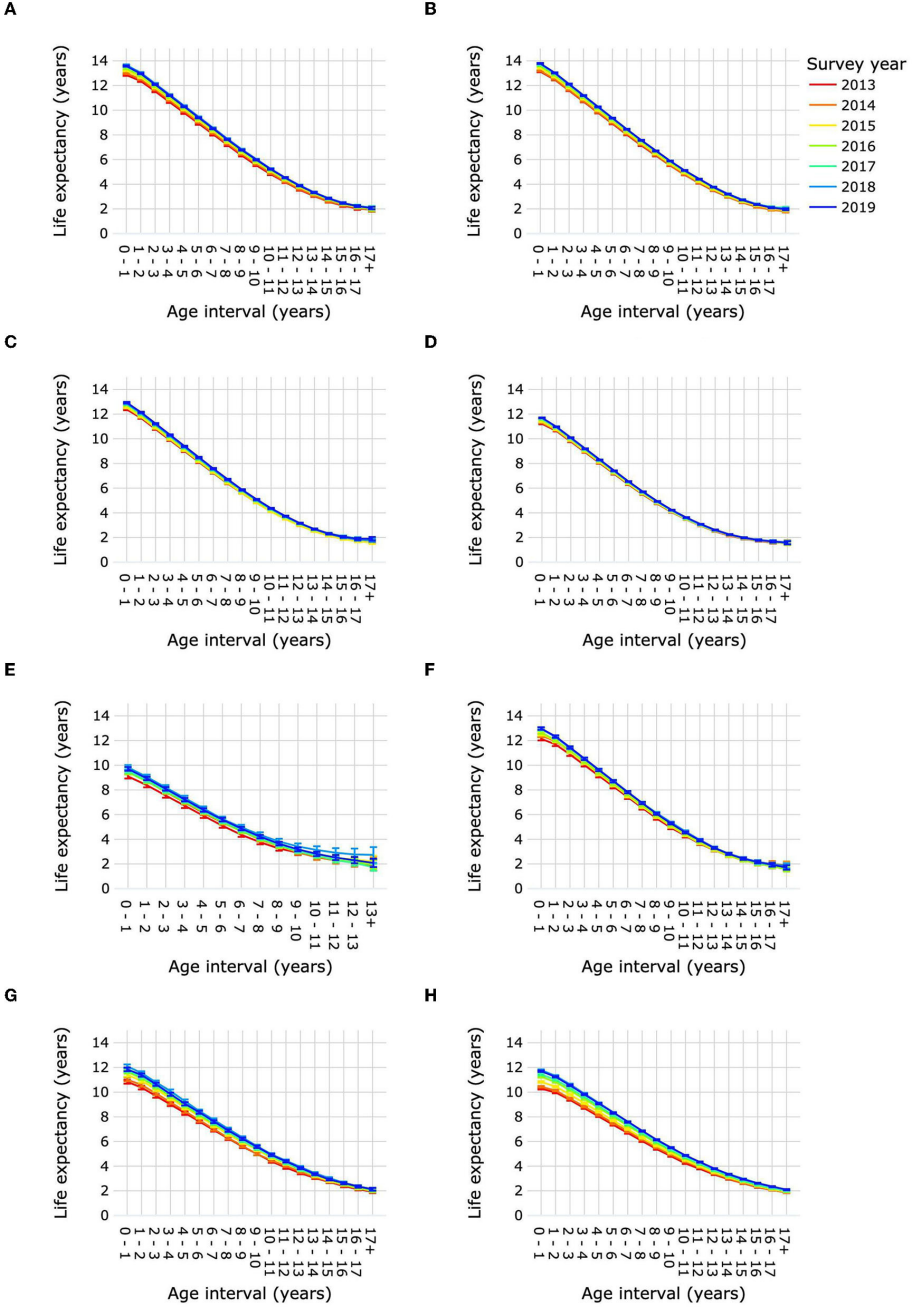
Life expectancies of purebred and mixed-breed dogs and cats by age interval and survey year. Life expectancies are shown for purebred dog size groups toy **(A)**, small **(B)**, medium **(C)**, large **(D)** and giant **(E)**, for mixed-breed dogs **(F)**, for purebred cats **(G)**, and for mixed-breed cats **(H)**. Error bars represent 95% confidence intervals.

Life expectancy for each age interval by survey year is presented in Figure 4 and Supplementary Table 8. For dogs, life expectancy was significantly higher in 2019 than in 2013 from the age interval 0–1 year to 15–16 years for toy and medium size groups, from 0–1 year to 16–17 years for small dogs, from 0–1 year to 13–14 years for large dogs, from 0–1 year to 8–9 years for giant dogs, and from 0–1 year to 11–12 years for mixed-breed dogs. Life expectancy was significantly higher in 2019 than in 2013 for mixed-breed cats at all age intervals, and for purebred cats from 0–1 year until 16–17 years.

### 3.4. Life expectancies by sex

**Figure 5:**
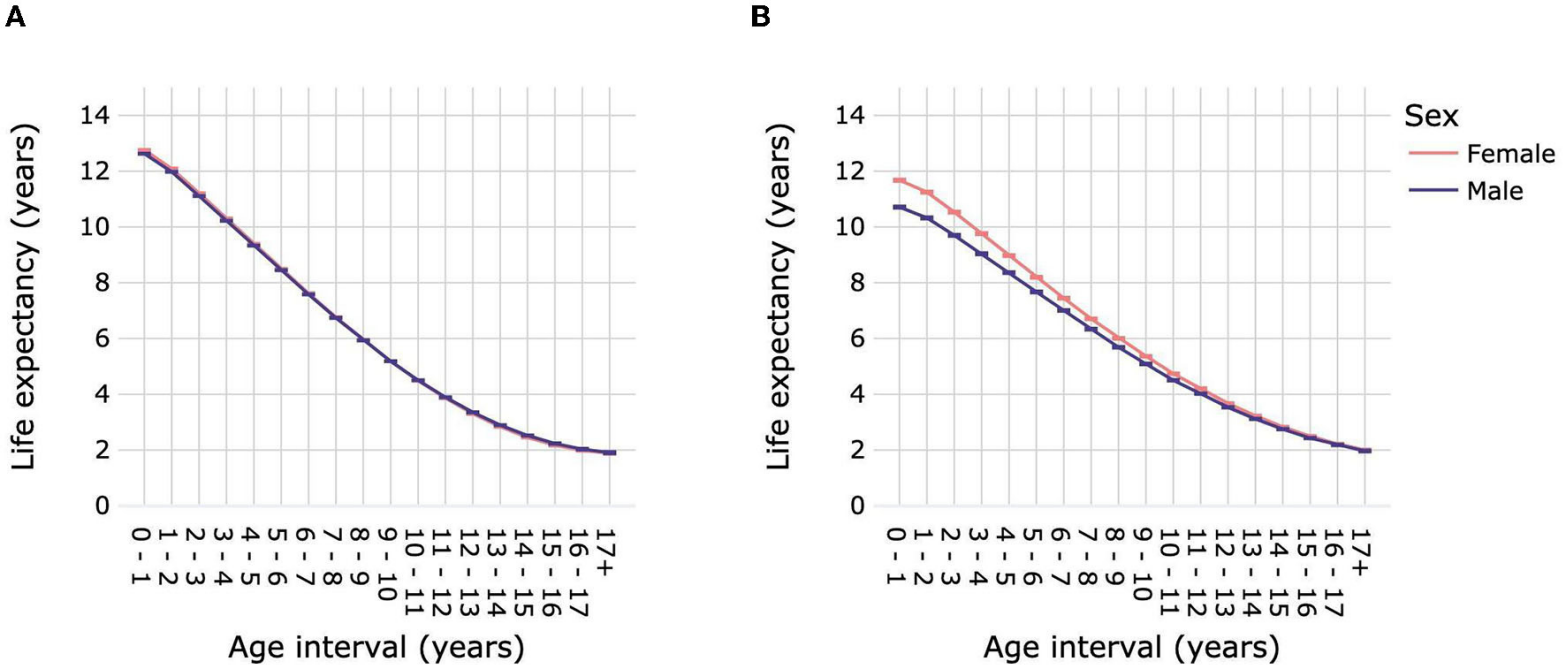
Life expectancies of dogs **(A)** and cats **(B)** by age interval and sex. Animal populations were all dogs and all cats regardless of size and breed. Error bars represent 95% confidence intervals.

**Figure 6:**
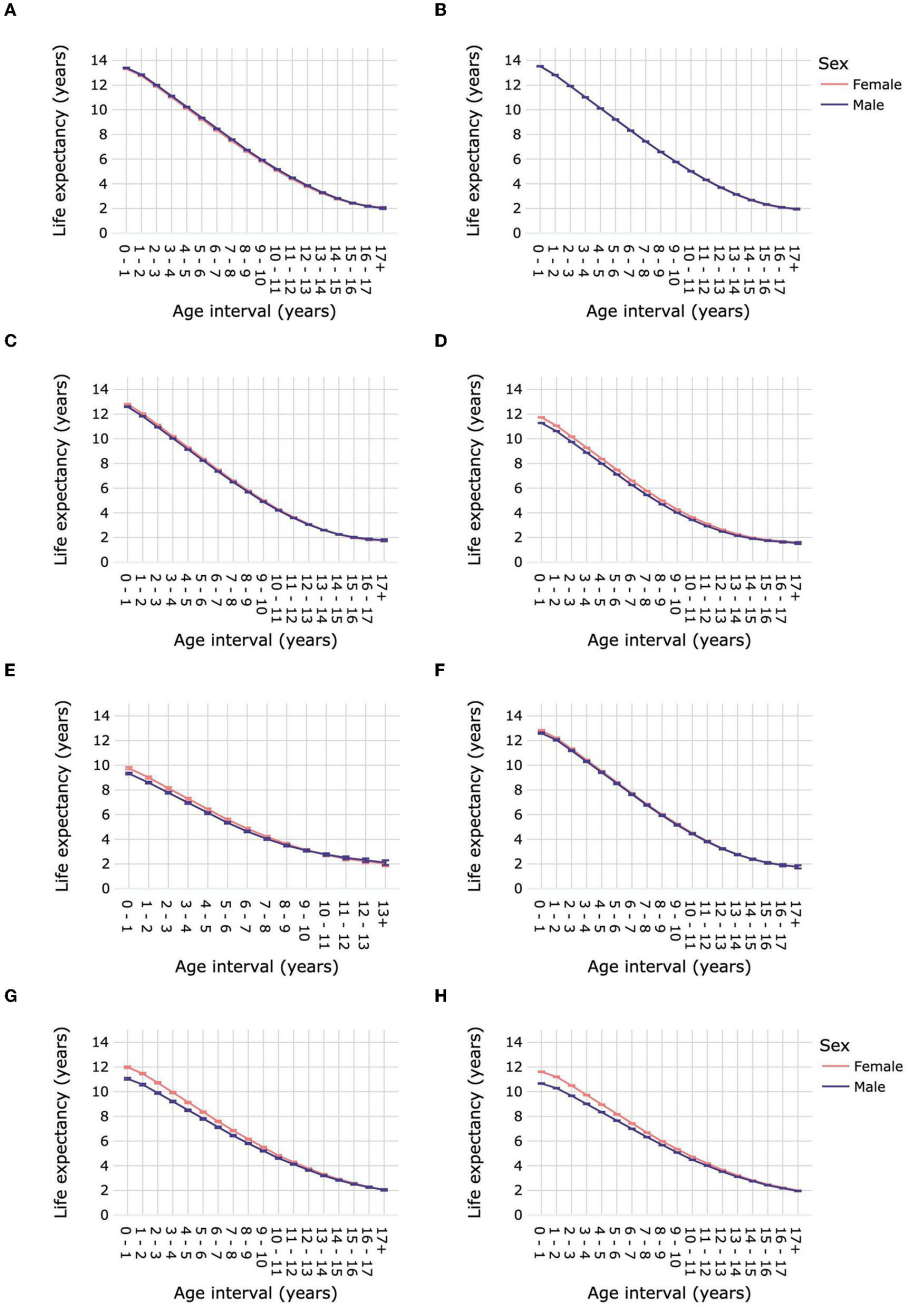
Life expectancies of purebred and mixed-breed dogs and cats by age interval and sex. Life expectancies are shown for purebred dog size groups toy **(A)**, small **(B)**, medium **(C)**, large **(D)** and giant **(E)**, for mixed-breed dogs **(F)**, for purebred cats **(G)**, and for mixed-breed cats **(H)**. Error bars represent 95% confidence intervals.

Female dogs had a slightly but significantly higher LE_birth_ than male dogs: 12.76 years (12.75–12.77) compared with 12.63 years (12.62–12.64) for males (Figure 5A; Supplementary Table 9). Life expectancy was significantly higher for female dogs over age intervals 0–1 year until 6–7 years compared with male dogs. From 11–12 years to 15–16 years male dogs had a significantly higher life expectancy than females. Sex differences in life expectancy varied according to dog size (Figure 6; Supplementary Table 10). Male toy dogs had a slightly but significantly higher LE_birth_ than female toy dogs [13.39 years (13.36–13.42) vs. 13.32 years (13.28–13.35)]. There were no significant sex differences in the life expectancy of small dogs in any age interval; LE_birth_ was 13.52 years for males (13.5–13.55) and 13.55 years (13.52–13.57) for females. In all other dog-size groups, females' LE_birth_ was significantly higher than that of males: medium 12.8 years (12.77–12.83) vs. 12.6 years (12.57–12.63), large 11.74 years (11.72–11.76) vs. 11.28 years (11.26–11.3), giant 9.76 years (9.66–9.85) vs. 9.33 years (9.24–9.41). Female mixed-breed dogs also had a significantly higher LE_birth_ than their male counterparts-−12.81 years (12.27–12.87) compared with 12.61 years (12.54–12.67).

Female cats had a significantly higher LE_birth_ than male cats, by ~1 year [11.68 years (11.65–11.71) vs. 10.72 years (10.68–10.75)] (Figure 5B; Supplementary Table 9). Differences in life expectancies between cat sexes progressively decreased until the age of 16–17 years, at which point they were similar, with overlapping confidence intervals. The LE_birth_ of purebred and mixed-breed cats were ~1 year higher than those of their male counterparts: purebred 11.98 years (11.91–12.06) vs. 11.05 years (10.97–11.14), mixed breed 11.62 (11.58–11.65) vs. 10.66 (10.63–10.7) (Figure 6; Supplementary Table 10).

### 3.5. Life expectancies by body condition score

**Figure 7:**
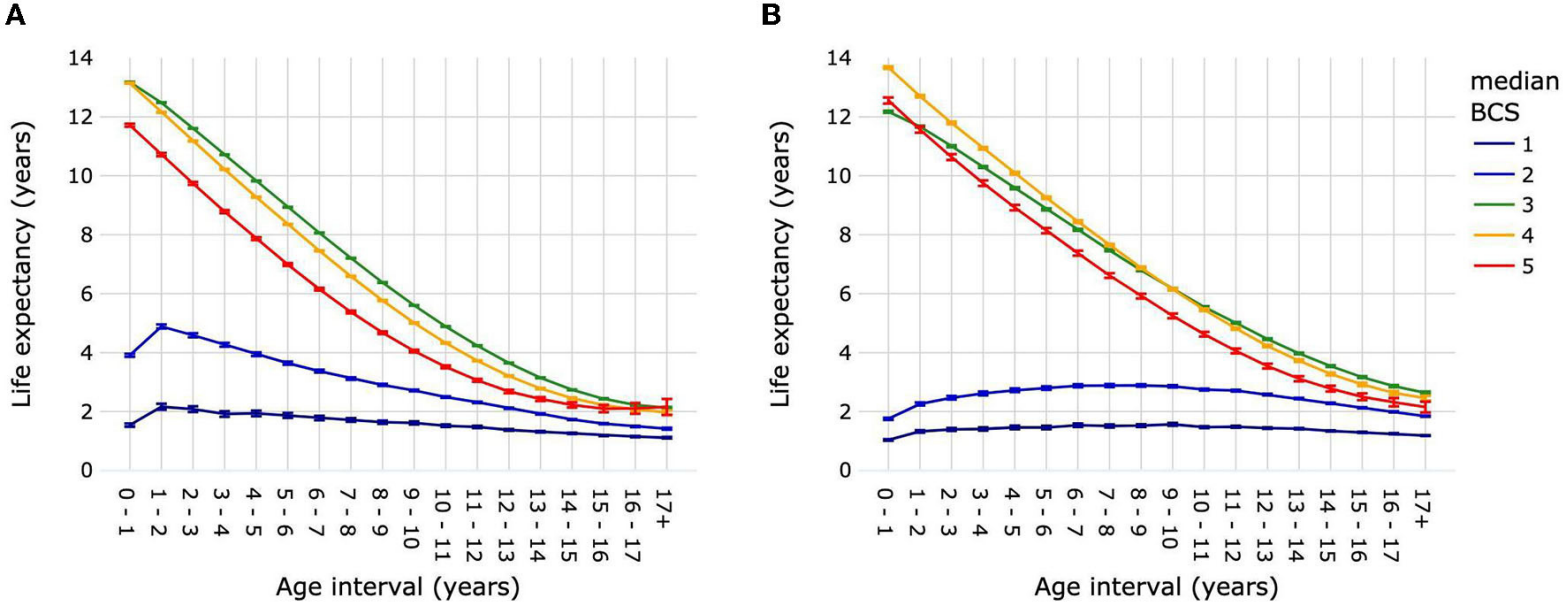
Life expectancies of dogs **(A)** and cats **(B)** by age interval and median life-time body condition score. Animal populations were all dogs and all cats regardless of size, breed and sex. Error bars represent 95% confidence intervals. Body condition scoring was on a 5-point scale from 1 = very thin to 5 = obese. BCS, body condition score.

Dogs with median BCS 3 and dogs with median BCS 4 had similar LE_birth_ of 13.18 years (13.16–13.19) and 13.14 years (13.12–13.16), respectively (Figure 7A; Supplementary Table 11). Life expectancy was significantly higher for dogs with median BCS 3 vs. median BCS 4 over the age interval 1–2 year until 17+ years. Dogs with median BCS 5 had a significantly lower LE_birth_ [11.71 years (11.66–11.77)] than dogs with median BCS 3 or median BCS 4. This continued to be the case for life expectancies in subsequent age intervals until 15–16 years, when the difference between median BCS 5 and median BCS 4 was no longer significant, while the difference between median BCS 5 and median BCS 3 remained significant until the age interval 17+ years. Dogs with median BCS 1 or median BCS 2 had significantly lower life expectancies compared with dogs of all other median BCS at all age intervals; LE_birth_ was 1.54 years (1.49–1.60) and 3.91 years (3.86–3.97), respectively. The difference in life expectancy between dogs with median BCS 1 and those with median BCS 2 diminished progressively after the age of 2 years, but remained significant until the age of ≥17 years.

Cats with median BCS 3 [12.18 years (12.14–12.21)] had a significantly lower LE_birth_ than cats with median BCS 4 [13.67 years (13.62–13.71)] or median BCS 5 [12.56 years (12.45–12.66)] (Figure 7B; Supplementary Table 12). Life expectancy continued to be lower in cats with median BCS 3 compared with median BCS 4 until the age interval of 9–10, when it was 6.16 years (6.13–6.19) vs. 6.15 years (6.11–6.19). After the age interval 10–11 and until 17+ years, median BCS 3 cats had a greater life expectancy than median BCS 4 cats. Life expectancies were significantly higher for cats of median BCS 5 compared with median BCS 3 only for age interval 0–1, after which they were significantly lower. The average life expectancy over all age intervals was ~1.39 years for cats with median BCS 1, and 2.49 years for cats with median BCS 2.

**Figure 8:**
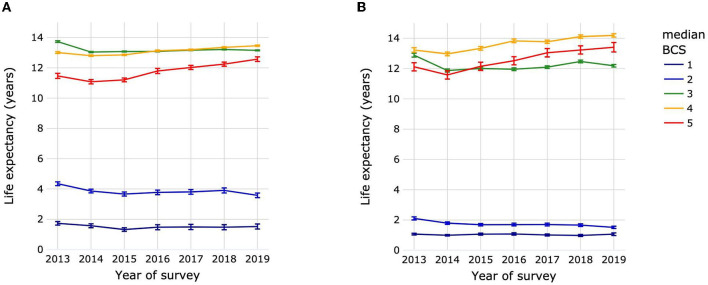
Life expectancies at birth of dogs **(A)** and cats **(B)** by year of survey and median life-time body condition score. Animal populations were all dogs and all cats regardless of size, breed and sex. Error bars represent 95% confidence intervals. Body condition scoring was on a 5-point scale from 1 = very thin to 5 = obese. BCS, body condition score.

The LE_birth_ of dogs by year of survey for median BCS 3 was 13.73 years (13.68–13.78) in 2013. This was significantly higher than for subsequent years, during which LE_birth_ increased slightly from 13.04 years (13.0–13.08) in 2014 to 13.15 years (13.12–13.18) in 2019, with few significant differences between years in this range (Figure 8A; Supplementary Table 13). The LE_birth_ of dogs with median BCS 5 also decreased between 2013 [11.47 years (11.3–11.63)] and 2014 [11.08 years (10.94–11.22)], but then increased by almost 1.5 to 12.57 years (12.42–12.72) in 2019. The mean LE_birth_ over all survey years for dogs with median BCS 1 was 1.51 years with no significant differences between years. For dogs with median BCS 3 or 4, the LE_birth_ was always significantly higher than that of dogs with BCS 5 regardless of the survey year.

The trends in LE_birth_ for cats were similar to those of dogs. Cats had a mean LE_birth_ over all survey years of 1.04 years with no significant differences between years in the survey (Figure 8B; Supplementary Table 13). The LE_birth_ for cats with median BCS 3 was 12.88 years (12.76–13.01) in 2013, dropping to 11.87 years (11.77–1.97) in 2014 before starting to increase slowly to 12.47 years (12.38–12.56) in 2018, falling back however to 12.19 years (12.1–12.27) in 2019. For cats with median BCS 5, there was an initial a drop in LE_birth_ from 12.12 years (11.85–12.39) in 2013 to 11.58 years (11.31–11.85) in 2014, followed by a 1.83 years increase in life expectancy between 2014 and 2019 [13.41 years (13.1–13.72)]. The LE_birth_ of cats with median BCS 4 was always significantly higher than that of cats with BCS 3 or 5 regardless of the survey year.

## 4. Discussion

This study generated annual life expectancy tables over a period of 7 years for dogs and cats aged from <1 to >17 years in the USA. Life expectancy tables were computed by survey year and by age in 1-year increments for each sex and each of five median BCS values.

### 4.1. Life expectancies overall and by size group or breed heritage

The overall LE_birth_ for dogs reported here [12.69 years (12.68–12.7)] was higher than that computed from the UK VetCompass™ dataset [11.23 years (11.19–11.27)] (15) and lower than that for dogs in two Japanese studies (13.7 years) (13, 14). The LE_birth_ in another Japanese study of dogs that died between 1981 and 1982 was only 8.3 years (12). This is more consistent with ours when consideration is given to the increase in life expectancy of dogs over time that we observed. The LE_birth_ by survey year increased by 1.3 months (0.93%) in each successive sampling year (Figure 2). A backwards projection estimates LE_birth_ to be 9.1 years for dogs in 1981. For comparative purposes with other studies, we also calculated the mean and median age of death of dogs; these were 10.55 and 11.47 years, respectively, which was within the range previously reported for dog populations comprising multiple breeds and both sexes (4–8).

Differences in overall life expectancy between studies might reflect in part different proportions of size categories, breeds and mixed-breed dogs. Japanese life tables by dog size showed the same order of decreasing LE_birth_ by size as observed in this study: small > toy > medium > large > giant (13). In an indirect comparison between the Japanese life expectancies and ours for size groups with the most comparable definitions, the LE_birth_ for small dogs was 14.2 years (14.0–14.4) compared with 13.53 years (13.52–13.55), respectively, and for toy dogs was 13.34 years (13.32–13.36) in japanese study and 13.36 years (13.33–13.38) in our study. The inverse relationship between dog size and longevity is well-described (42), however, even within a size category, the breed composition may affect the life expectancy computation. For example, in the UK, the two highest life expectancies by breed were for the Jack Russell Terrier (small size) and the Yorkshire terrier (toy size), but the Border Collie (medium size) ranked third and the Chihuahua (toy) ranked below this in 15th place (15).

Multiple other differences between studies might also explain differences in life expectancies: population size and source, case ascertainment, husbandry and veterinary practices of the countries concerned and the dates the data relate to. The life expectancy of insured dogs might be enhanced compared with uninsured dogs by both a greater willingness of owners to seek veterinary care and by representation of breeds predisposed to health conditions that incur higher insurance premiums. Frequency of veterinary visits could indicate the presence of a chronic health condition, but might also reflect participation in a wellness program of routine preventative veterinary care, including vaccination. The study country is likely to influence these and other factors. Dogs in the USA will have genetically different breeding lines from the UK and Japan. Broad cultural differences in human populations can affect the lifestyle, environment, and nutrition of their pets, as well as owners' access to and attitude toward veterinary interventions. In terms of methodological variations in life expectancy studies, the reliability of information from veterinary medical records such as used in this study may be different to that from owners, as used in the most recent Japanese cemetery survey (14). However, veterinary records will of course exclude all pets never visiting a veterinary clinic, which is likely to include many puppies dying between birth and weaning [e.g., 9% of purebred puppies in Norway (43)].

In the only life expectancy tables we found for cats the LE_birth_ was only 4.2 years, which is approximately 7 years less than in the current study (16). However, the data were from 1981 and 1982. In our study, life expectancy increased on average by 2.6 months (2.01%) each successive sampling year from 2013 to 2019 (Figure 2), and a backwards projection estimates LE_birth_ to be 5.42 years for cats in 1981.

Age-at-death data are also scarce for cat populations comprising multiple breeds and or mixed-breed cats. The median age-at-death of cats between 2009 and 2012 in a UK study was 14.0 years (IQR: 9.1–17.0) (9). This is substantially higher than for our population, which we calculated for comparative purposes to be 12.18 years (IQR: 6.7–15.6). It is difficult to attribute this difference to a specific factor or combination of factors, although the country context is likely to have played a role. Many of the inter-study differences in populations, methodology, pet husbandry and veterinary care that were discussed for dogs might also have contributed to differences in cat data. Another factor to consider is that in the USA, although the owned cat population exceeds the owned dog population, the mean number of veterinary visits per household per year was estimated to be 2.4 for dogs and 1.3 for cats in 2016, and the percentage of yearly routine or preventive care visits was 78.8% for dogs compared with 47.2% for cats (44). Our study population contained proportionately fewer cats than dogs between the ages of 3 and 11 years, and proportionally more from the age of 12 years, when chronic conditions and conditions of old age will be most apparent. The threshold of owner-perceived severity of illness that will prompt a veterinary visit is higher for cats than dogs. Taking these factors together may indicate that our study underestimated life expectancy of cats.

The concept of hybrid vigor has led to a perception that mixed-breed pets live longer than purebred pets, and indeed, this has been demonstrated for size-matched dogs (45). Our study was not designed to test this hypothesis, and life expectancies for mixed-breed dogs of any size were similar to those of medium-sized purebred dogs. In contrast, purebred cats had consistently longer life expectancies than mixed-breed cats. This might be explained if a greater percentage of purebred cats were kept indoors, where they would be at a lower risk of fights and accidents (46).

Overall, for both dogs and cats, LE_birth_ progressively increased except for a small decrease in 2019, which was significant for cats but not for dogs. This might be explained by the impact of the COVID-19 pandemic in 2020 and 2021. During this time in the USA, veterinary practices were subject to emergency regulations, and practice visits were curtailed in many areas to “essential” services only, while telemedicine was actively encouraged (3, 47). Pet owners were therefore likely to have made veterinary visits only for seriously ill or injured animals, which would tend to have decreased the survivor population but not affected the deceased population. Visits in 2020 and 2021, from which the 2019 survival population was estimated, probably impacted the return rate of visits within 2 years (Supplementary Table 2). Indeed, the return rate was increasing between 2013 (74.8% for dogs, 61.9% for cats) and 2017 (77.6% for dogs, 65.4% for cats), but was meaningfully lower in 2019 (75.9% for dogs and 62.5% for cats) compared to 2018 (77% for dogs and 65.1% for cats). Such a reduction would have artificially increased the number of excluded animals. Extending the follow-up to 2022 may have allowed a more accurate estimation of survivors in 2019.

### 4.2. Life expectancies by sex

Although female dogs had a significantly higher LE_birth_ than male dogs, the difference was small (~1.5 months) and did not persist in older age groups. A 4-month difference was shown between the sexes in a UK dog population [LE_birth_ 11.41 years (11.35–11.47) for females vs. 11.07 years (11.01–11.13) for males], and this also declined with increasing age.

The finding that LE_birth_ for female cats was 1 year higher compared with that for male cats reflected a higher death rate for males between the ages of 1 and 9 years. It was hypothesized that these sex differences were due to the higher probability of infection with feline leukemia virus and feline immunodeficiency virus (48, 49), and also a higher probability of lower urinary tract obstruction, which occurs almost exclusively in males (50). Accidents and fights are also more common for males between 1 and 2 years of age than for females. Neoplasia and other diseases associated with chronic retroviral infection, and complications of urethral obstruction can be fatal (49, 50). A preliminary investigation of the causes of death for male vs. female cats in the current study's database found the main contributor between birth and 10 years was urinary tract obstruction, which accounted for ~15% of male deaths in the 3–4 years age interval (data not presented).

### 4.3. Life expectancies by body condition score

Our finding that obese dogs (median BCS 5) had 1.5 years lower LE_birth_ than dogs with median BCS closer to the ideal (median BCS 3) is consistent with another primarily USA analysis that found overweight and obesity to be associated with a higher instantaneous risk of death and a reduced life span (20). In that study, the hazard ratio for death in overweight compared with normal weight dogs varied by breed from 1.35 (99.79% CI: 1.05–1.73) to 2.86 (99.79% CI: 2.14–3.83) (20). We observed that the impact of overweight or obese body condition on life expectancy was lower in the oldest dogs. It is hypothesized that this could be the influence of the “obesity paradox”, whereby a higher body condition score or increase in weight may have some protective survival effect in dogs (and other animals and humans) with chronic disease, such as chronic kidney disease (51) and heart failure (52).

Obesity in dogs is associated with a wide diversity of disease types, ranging for example from metabolic such as diabetes mellitus (26), to orthopedic (19), and cancer (53), some of which might impact lifespan. In terms of the relationship between body condition and disease, obesity is likely to be a causative risk factor for many of these conditions e.g., diabetes, but it can be a consequence of some other diseases such as hyperadrenocorticism and hypothyroidism (54–56). Obesity could also be an unrelated comorbidity that exacerbates the severity and/or prognosis of another condition, or contributes to a decision on euthanasia. It is not possible to attribute adiposity in our study as a cause of reduced LE_birth_, but since the database included both healthy and diseased animals it is feasible that it played an indirect role through disease association.

A further consideration is that we used the median BCS over a pet's medical history in the database to reflect its weight status over life. This approach could lead to misinterpretation in cases where an age-related disease affected BCS. For example, if an animal with a BCS of 5 in the first third of its life contracted a disease in the second third of its life that led to a reduction in BCS to BCS 3, and with disease progression this declined to BCS 1 in the last third of its life, the animal would nevertheless be analyzed in the median BCS 3 category.

Less than ideal median BCS (1 and 2) was associated with much lower life expectancy than an ideal or obese median BCS. In adult dogs, underweight is a general signalment for serious illness or a poor prognostic factor for diagnosed disease. For example, low BCS in dogs with chronic enteropathy is a predictor for lack of response to treatment and death (57). Underweight in young animals is perceived as a failure to thrive, and low birth weight puppies have an increased risk of neonatal death (58). In our population, life expectancy increased significantly between the age interval 0–1 year and 1–2 years for dogs with median BCS 1, and, to a lesser extent, dogs with median BCS 2.

Similar to dogs, overweight and obesity in cats are associated with a wide range of disorders, including endocrine and lipid metabolism disorders, neoplasia, lower urinary tract disease, gastrointestinal disease and dermatoses (30, 59). However, in contrast to dogs, overweight cats (median BCS 4) had a higher LE_birth_ than cats of ideal body condition (median BCS 3) as well as obese cats (Figure 4); the difference persisted although reduced with increasing age. This is not so surprising in the light of an Australian study that found that cats with a maximum BCS of 6–8 on a 9-point scale (i.e., overweight to obese) had higher survival over time compared with cats with maximum BCS 3–5 or BCS 9 (obese) (21). The authors hypothesized that having a BCS of 6–8 might have the least negative effects on lifespan, or that it might be associated with owners who provide better care for their pets. The relationship between BCS and survival is complex. The same study found that the age at which maximum BCS was reached influenced the relative effects of different BCS on lifespan. A factor that might be relevant to this is the association of age with susceptibility to different types of diseases, including acute or chronic, easily treatable or life-threatening. For example, risk of serious viral diseases such as feline infectious peritonitis is higher in younger cats (60), while diabetes mellitus, cardiovascular disease and feline lymphoma are associated with old age (61–63). Finally, the same limitations mentioned above of using median BCS in dogs apply to cats.

As for dogs, underweight cats had a lower life expectancy at all ages than cats with ideal, overweight or obese median BCS. However, life expectancy in underweight cats did not decline; cats of median BCS 1 consistently had almost 1 year of life expectancy and cats of median BCS 2 consistently had ~2 years of life expectancy. A range of interacting factors might influence the life expectancy of cats with low median BCS in different ways. For example, weight loss is one sign of chronic kidney disease in cats (64); it is often present before CKD has been diagnosed, and lower bodyweight is associated with reduced survival time from diagnosis (65). It is plausible that owners delay seeking veterinary care for weight loss in cats until CKD is advanced and has a poor prognosis. In Banfield medical records, 52% of dogs and cats diagnosed with CKD die within 18 months and 10% are euthanized within 18 months (unpublished data).

For both dogs and cats, the LE_birth_ of animals with median BCS 3 was quite stable over survey years, while for animals with median BCS 5 a relatively large increase of LE_birth_ was observed between 2014 and 2019: 1.51 years for dogs and 1.93 years for cats. Although these findings may be related to advances in veterinary medicine that would allow obese dogs to live longer, they also raise new questions about potential changes in veterinarians' perception of, and attitudes toward obesity. It is possible that a dog considered to be BCS 5 in 2014 would be considered to be BCS 3 according to perceptions in 2019. Increasing awareness of obesity and its health implications in pets could also have driven greater proactivity in interventions to treat obesity-associated diseases.

Our clinical dataset relied upon owners visiting a veterinarian, and the animal having a recorded BCS. There may be different thresholds of bodyweight loss or bodyweight gain that would prompt owners to seek veterinary advice. Delaying a visit until a dog or cat has reached an extreme BCS will worsen the prognosis for the underlying etiology. The decision of an owner to opt for the euthanasia of their dog or cat might also vary by BCS and thereby impact life expectancy. Pet lifestyle can impact survival for medical and non-medical reasons. An indoor lifestyle is associated with feline obesity at 1 year of age (66), and outdoor access poses a greater risk of road traffic accidents (46).

### 4.4. Strengths and limitations of the study

This study benefited from a large dataset of 8.9 million dogs and 2.4 million cats, which is more than 10-fold greater than the dataset used to generate life expectancy tables for Japanese dogs (13) and 100-fold greater than the dataset for life expectancy study of dogs in the UK. The database was large enough for a sub-analysis by BCS, and CIs remained modest with the exception of LE_birth_ for cats with BCS 5, and life expectancy for dogs and cats with BCS 5 over the age interval 14–15 years, likely due to the small number of obese animals in these age intervals.

Limitations that should be considered include the retrospective nature of the study design, a degree of uncertainty of pet age for dogs and cats acquired as rescued animals or adopted from animal shelters, and the inherent limitations of body condition scoring. In clinical practice body condition scoring is routinely used to estimate adiposity and weight status, scoring systems have been validated against measurement of body fat by dual-energy X-ray absorptionmetry (67–72) and good reproducibility of scoring between different veterinarians has been reported (73). Nevertheless, body condition scoring does have the disadvantage of being a subjective and semi-quantitative measure, and there is a risk of inconsistencies arising between numerous veterinarians scoring body condition over a number of years. A general shift in veterinarians' perception of obesity over time could also impact the median BCS as previously noted. In this study, medical records were only available for a 9-year period rather than over each pets' lifetime. We used median BCS between 2013 and 2022 to address the complication of changes in BCS over time and to focus on pets that had BCS data covering the majority of their life. A limitation of this was that the number of visits and BCS evaluations was different between individuals in any survey year and across all survey years. The method could not account for the duration of overweight or underweight status, or the age at which the median BCS occurred. Other drawbacks to using median BCS have been discussed above. Another point to note is that although it is used in practice in young animals, the BCS system has not been validated in puppies and kittens. The accuracy of body condition scoring in young and aged animals may be reduced because it does not account for “natural” differences and changes in body composition with age.

The intentional omission of some aspects of life expectancy in our analyses might be regarded as limitations. We decided not to analyze any effect of neuter status, because ~90% of owned dogs and cats in the USA are neutered, and this is reflected in the clinical Banfield population. Differences in life expectancies between breeds within body size were not captured. The intent was to maintain accuracy in life expectancy tables while recognizing the large size differences between breeds. As population numbers were low for many of the more than 300 breeds in our dataset, we favored statistical accuracy over breed differences. Parallel analyses of health status, recorded causality of death, or reason for euthanasia were beyond the scope of this study and this limited the interpretation of differences between subpopulations.

Most pets that visit Banfield Pet Hospitals have veterinary care as part of an Optimal Wellness Plan, which is a suite of pre-paid services focused on preventive health care, routine diagnostic testing, comprehensive physical examination, and unlimited clinic visits. Wellness Plans could result in a pet being seen more frequently and having an earlier diagnosis and treatment of chronic conditions; they might also drive a proactive reduction in risk factors for disease and improvement in prognosis for disease at diagnosis. Conversely, the use of medical records results in missed visits by animals never presented to veterinary clinics.

A major statistical consideration with databases involving millions of individuals is that tests are often highly significant. A widely used method for assessing the standard error of life expectancy is to use the variance of the conditional probability of death. To address the issue of having such large populations, instead of implementing this method we used Monte Carlo simulations to sample dead populations and recalculated life expectancies 1.10^6^ times. This mitigated the issue, but statistical significances were still high whatever significance level. A complementary strategy would have been to sample the mid-year population in the Monte Carlo simulation.

The analyses presented do not extend to estimations of disability free life expectancy, healthy life expectancy or healthy life years lost. A method has been developed for this (74) and applied to life expectancy data from dogs in the UK (15) and Japan (13) for different breeds and cross-breed dogs (75). The Banfield database provides additional opportunities to explore healthy life expectancy and specific disease-associated life expectancy in future work.

### 4.5. Summary and closing remarks

To our knowledge this is the first study to generate life expectancy tables for dogs and cats in the USA and the first study to present these by median BCS. Some of the main findings were expected based on direct and indirect evidence in the literature, namely decreased life expectancy for the largest sizes of dogs compared to the smallest, increased life expectancy of female versus male dogs and cats, significantly lower life expectancy at all ages for underweight dogs and cats compared to those with an ideal, overweight or obese median BCS, and a progressive increase in LE_birth_ for both species between 2013 and 2018. Unexpected findings included the relatively constant life expectancy of underweight cats at all ages, and the higher life expectancy of overweight cats compared with cats that were ideal median BCS. Further study is needed to elucidate apparently complex, interrelated and dynamic relationships between median BCS and life expectancy.

The life expectancy tables presented provide valuable information for practicing veterinarians and a foundation for research hypotheses. A better knowledge of life expectancy is important at the population level as a broad indicator of the general health status of the pet population, reflecting owner attitudes, the accessibility of veterinary care, and advances in veterinary care including preventive medicine. At the level of the individual owner, these life expectancy tables may help inform the choice of species and breed size. Life expectancy tables are especially relevant for adopters of adult animals who need to understand the likely duration of their commitment. Quantification of the reduction in life expectancy associated with overweight and/or obesity is a vital step toward making proactive bodyweight management a healthcare priority for veterinarians and owners. Furthermore, this work is a stepping-stone to the generation of disease-associated life expectancy tables.

## Data availability statement

The data analyzed in this study is subject to the following licenses/restrictions: Raw data cannot be shared for legal and privacy restrictions. Requests to access these datasets should be directed to mathieu.montoya@royalcanin.com.

## Author contributions

MM contributed to the study design, data processing and interpretation of the results, performed the statistical analyses, wrote the first draft of the manuscript, and revised the intellectual content of subsequent drafts. FA contributed substantially to the design of the study and the statistical analysis work and critically reviewed the intellectual content of the manuscript. JM, NS, VB, HC, and M-AH contributed to data processing and interpretation of the results and critically reviewed the intellectual content of the manuscript. All authors have approved the final content of the manuscript for publication.

## References

[1] Gompertz B. XXIV. On the nature of the function expressive of the law of human mortality, and on a new mode of determining the value of life contingencies. Philos Transact R Soc London. (1825) 115:513–83. 10.1098/rstl.1825.0026 PMC4360127 25750242

[2] Office for National Statistics. Life Expectancy Releases and Their Different Uses. (2018). Available from: https://www.ons.gov.uk/peoplepopulationandcommunity/healthandsocialcare/healthandlifeexpectancies/articles/lifeexpectancyreleasesandtheirdifferentuses/2018-12-17 (acessed July 11, 2022).

[3] American Veterinary Medical Association. Covid 19. Available from: https://www.avma.org/resources-tools/one-health/covid-19 (acessed July 10, 2022).

[4] Lewis TW Wiles BM Llewellyn-Zaidi AM Evans KM O'Neill DG. Longevity and mortality in Kennel Club registered dog breeds in the UK in 2014. Canine Genet Epidemiol. (2018) 5:10. 10.1186/s40575-018-0066-8 30349728 PMC6191922

[5] Adams VJ Evans KM Sampson J Wood JL. Methods and mortality results of a health survey of purebred dogs in the UK. J Small Anim Pract. (2010) 51:512–24. 10.1111/j.1748-5827.2010.00974.x 21029096

[6] Proschowsky HF Rugbjerg H Ersbøll AK. Mortality of purebred and mixed-breed dogs in Denmark. Prev Vet Med. (2003) 58:63–74. 10.1016/S0167-5877(03)00010-2 12628771

[7] Michell AR. Longevity of British breeds of dog and its relationships with sex, size, cardiovascular variables and disease. Vet Rec. (1999) 145:625–9. 10.1136/vr.145.22.625 10619607

[8] O'Neill DG Church DB McGreevy PD Thomson PC Brodbelt DC. Longevity and mortality of owned dogs in England. Vet J. (2013) 198:638–43. 10.1016/j.tvjl.2013.09.020 24206631

[9] O'Neill DG Church DB McGreevy PD Thomson PC Brodbelt DC. Longevity and mortality of cats attending primary care veterinary practices in England. J Feline Med Surg. (2015) 17:125–33. 10.1177/1098612X14536176 24925771 PMC10816413

[10] Urfer SR Wang M Yang M Lund EM Lefebvre SL. Risk factors associated with lifespan in pet dogs evaluated in primary care veterinary hospitals. J Am Anim Hosp Assoc. (2019) 55:130–7. 10.5326/JAAHA-MS-6763 30870610

[11] Egenvall A Nødtvedt A Häggström J Ström Holst B Möller L Bonnett BN. Mortality of life-insured Swedish cats during 1999–2006: age, breed, sex, and diagnosis. J Vet Intern Med. (2009) 23:1175–83. 10.1111/j.1939-1676.2009.0396.x 19780926 PMC7167180

[12] Hayashidani H Omi Y Ogawa M Fukutomi K. Epidemiological studies on the expectation of life for dogs computed from animal cemetery records. Nihon Juigaku Zasshi. (1988) 50:1003–8. 10.1292/jvms1939.50.1003 3199609

[13] Inoue M Hasegawa A Hosoi Y Sugiura K. A current life table and causes of death for insured dogs in Japan. Prev Vet Med. (2015) 120:210–8. 10.1016/j.prevetmed.2015.03.018 25896026

[14] Inoue M Kwan NCL Sugiura K. Estimating the life expectancy of companion dogs in Japan using pet cemetery data. J Vet Med Sci. (2018) 80:1153–8. 10.1292/jvms.17-0384 29798968 PMC6068313

[15] Teng KT Brodbelt DC Pegram C Church DB O'Neill DG. Life tables of annual life expectancy and mortality for companion dogs in the United Kingdom. Sci Rep. (2022) 12:6415. 10.1038/s41598-022-10341-6 35484374 PMC9050668

[16] Hayashidani H Omi Y Ogawa M Fukutomi K. Epidemiological studies on the expectation of life for cats computed from animal cemetery records. Nihon Juigaku Zasshi. (1989) 51:905–8. 10.1292/jvms1939.51.905 2607740

[17] Urfer SR. Right censored data (‘cohort bias') in veterinary life span studies. Vet Rec. (2008) 163:457–8. 10.1136/vr.163.15.457 18849580

[18] Paynter AN Dunbar MD Creevy KE Ruple A. Veterinary big data: when data goes to the dogs. Animals. (2021) 11:1872. 10.3390/ani11071872 34201681 PMC8300140

[19] German AJ. The growing problem of obesity in dogs and cats. J Nutr. (2006) 136:1940S–6S. 10.1093/jn/136.7.1940S 16772464

[20] Salt C Morris PJ Wilson D Lund EM German AJ. Association between life span and body condition in neutered client-owned dogs. J Vet Intern Med. (2019) 33:89–99. 10.1111/jvim.15367 30548336 PMC6335446

[21] Teng KT McGreevy PD Toribio JL Raubenheimer D Kendall K Dhand NK. Strong associations of nine-point body condition scoring with survival and lifespan in cats. J Feline Med Surg. (2018) 20:1110–8. 10.1177/1098612X17752198 29393723 PMC11104206

[22] Bach JF Rozanski EA Bedenice D Chan DL Freeman LM Lofgren JL. Association of expiratory airway dysfunction with marked obesity in healthy adult dogs. Am J Vet Res. (2007) 68:670–5. 10.2460/ajvr.68.6.670 17542702

[23] Chiang CF Villaverde C Chang WC Fascetti AJ Larsen JA. Prevalence, risk factors, and disease associations of overweight and obesity in cats that visited the veterinary medical teaching hospital at the University of California, Davis from January 2006 to December 2015. Top Comp Anim Med. (2022) 47:100620. 10.1016/j.tcam.2021.100620 34936906

[24] Henegar JR Bigler SA Henegar LK Tyagi SC Hall JE. Functional and structural changes in the kidney in the early stages of obesity. J Am Soc Nephrol. (2001) 12:1211–7. 10.1681/ASN.V1261211 11373344

[25] Lund EM Armstrong PJ Kirk CA Klausmer JS. Prevalence and risk factors for obesity in adult cats from private US veterinary practices. Int J Appl Res Vet Med. (2005) 3:88–96.

[26] Lund EM Armstrong PJ Kirk CA Klausner JS. Prevalence and risk factors for obesity in adult dogs from private US veterinary practices. Int J Appl Res Vet Med. (2006) 4:177–86.

[27] Manens J Bolognin M Bernaerts F Diez M Kirschvink N Clercx C. Effects of obesity on lung function and airway reactivity in healthy dogs. Vet J. (2012) 193:217–21. 10.1016/j.tvjl.2011.10.013 22099184

[28] Parker VJ Orcutt E Love L. Pathophysiology of obesity: comorbidities and anesthetic considerations. In:Cline MG Murphy M, editors. Obesity in the Dog and Cat. 1st ed. Boca Raton, FL: CRC Press (2019). p. 40–61.

[29] Tropf M Nelson OL Lee PM Weng HY. Cardiac and metabolic variables in obese dogs. J Vet Intern Med. (2017) 31:1000–7. 10.1111/jvim.14775 28608635 PMC5508341

[30] Teng KT McGreevy PD Toribio J Raubenheimer D Kendall K Dhand NK. Associations of body condition score with health conditions related to overweight and obesity in cats. J Small Anim Pract. (2018) 59:603–15. 10.1111/jsap.12905 30033652

[31] Tvarijonaviciute A Ceron JJ Holden SL Cuthbertson DJ Biourge V Morris PJ. Obesity-related metabolic dysfunction in dogs: a comparison with human metabolic syndrome. BMC Vet Res. (2012) 8:147. 10.1186/1746-6148-8-147 22929809 PMC3514388

[32] Sullivan DF A. single index of mortality and morbidity. HSMHA Health Rep. (1971) 86:347–54. 10.2307/4594169 5554262 PMC1937122

[33] Imai K Soneji S. On the estimation of disability-free life expectancy: sullivan's method and its extension. J Am Stat Assoc. (2007) 102:1199–211. 10.1198/016214507000000040 26279593 PMC4533834

[34] Jagger C. Can we live longer, healthier lives? In:Zeng YI Crimmins EM Carrière Y Robine J-M, editors. Longer Life and Healthy Aging. Dordrecht: Springer (2006). p. 7–22.

[35] Lopez AD Ahmad OB Guillot M Inoue M Ferguson BD Salomon JA. Life tables for 191 countries for 2000: data, methods, results. In:Murray CJL Evans DB, editors. Health Systems Performance Assessment: Debates, Methods and Empiricism. Geneva: World Health Organization (2001). p. 335.

[36] Silcocks PBS Jenner DA Reza R. Life expectancy as a summary of mortality in a population: Statistical considerations and suitability for use by health authorities. J Epidemiol Community Health. (2001) 55:38–43. 10.1136/jech.55.1.38 11112949 PMC1731769

[37] Toson B Baker A. Life Expectancy at Birth: Methodological Options for Small Populations. Newport: The Office of National Statistics (2003). 27 p

[38] Wolfson MC. Health-adjusted life expectancy. Health Rep. (1996) 8:41–5. 8844180

[39] Salomon JA Murray CJ. Compositional models for mortality by age, sex and cause. In: Global Programme on Evidence for Health Policy GPE Discussion Paper Series: No 11. Geneva: World Health Organization (2001).

[40] Shkolnikov VM Andreev EM McKee M Leon DA. Components and possible determinants of the decrease in Russian mortality in 2004-2010. Demogr Res. (2010) 28:917–50. 10.4054/DemRes.2013.28.32

[41] Antolin P. Longevity risk and Private Pensions. OECD Working Papers on Insurance and Private Pensions. Paris: OECD Publishing (2007). 28 p.

[42] Kraus C Pavard S Promislow DE. The size-life span trade-off decomposed: why large dogs die young. Am Nat. (2013) 181:492–505. 10.1086/669665 23535614

[43] Tønnessen R Borge KS Nødtvedt A Indrebø A. Canine perinatal mortality: a cohort study of 224 breeds. Theriogenology. (2012) 77:1788–801. 10.1016/j.theriogenology.2011.12.023 22365700

[44] AVMA. Pet Ownership & Demographics Sourcebook: 2017-2018 edition. Washington, DC: American Veterinary Medical Association (2017).

[45] Yordy J Kraus C Hayward JJ White ME Shannon LM Creevy KE. Body size, inbreeding, and lifespan in domestic dogs. Conserv Genet. (2020) 21:137–48. 10.1007/s10592-019-01240-x 32607099 PMC7326369

[46] Tan SML Stellato AC Niel L. Uncontrolled outdoor access for cats: an assessment of risks and benefits. Animals. (2020) 10:258. 10.3390/ani10020258 32041155 PMC7070728

[47] US Food & Drug Administration. Coronavirus (COVID-19) Update: FDA Helps Facilitate Veterinary Telemedicine During Pandemic. (2020). Available from: https://www.fda.gov/news-events/press-announcements/coronavirus-covid-19-update-fda-helps-facilitate-veterinary-telemedicine-during-pandemic (accessed January 27, 2022).

[48] Chhetri BK Berke O Pearl DL Bienzle D. Comparison of risk factors for seropositivity to feline immunodeficiency virus and feline leukemia virus among cats: a case-case study. BMC Vet Res. (2015) 11:30. 10.1186/s12917-015-0339-3 25889006 PMC4332748

[49] Little S Levy J Hartmann K Hofmann-Lehmann R Hosie M Olah G. 2020 AAFP feline retrovirus testing and management guidelines. J Feline Med Surg. (2020) 22:5–30. 10.1177/1098612X19895940 31916872 PMC11135720

[50] Segev G Livne H Ranen E Lavy E. Urethral obstruction in cats: predisposing factors, clinical, clinicopathological characteristics and prognosis. J Feline Med Surg. (2011) 13:101–8. 10.1016/j.jfms.2010.10.006 21145768 PMC10822313

[51] Parker VJ Freeman LM. Association between body condition and survival in dogs with acquired chronic kidney disease. J Vet Intern Med. (2011) 25:1306–11. 10.1111/j.1939-1676.2011.00805.x 22092621

[52] Slupe JL Freeman LM Rush JE. Association of body weight and body condition with survival in dogs with heart failure. J Vet Intern Med. (2008) 22:561–5. 10.1111/j.1939-1676.2008.0071.x 18466257

[53] Marchi PH Vendramini TH Perini MP Zafalon RV Amaral AR Ochamotto VA. Obesity, inflammation, and cancer in dogs: review and perspectives. Front Vet Sci. (2022) 9:1004122. 10.3389/fvets.2022.1004122 36262532 PMC9573962

[54] Cho KD Paek J Kang JH Chang D Na KJ Yang MP. Serum adipokine concentrations in dogs with naturally occurring pituitary-dependent hyperadrenocorticism. J Vet Intern Med. (2014) 28:429–36. 10.1111/jvim.12270 24372863 PMC4857983

[55] German A. Obesity in companion animals. Int Pract. (2010) 32:42–50. 10.1136/inp.b5665

[56] Scott-Moncrieff JC. Clinical signs and concurrent diseases of hypothyroidism in dogs and cats. Vet Clin North Am Small Anim Pract. (2007) 37:709–22, vi. 10.1016/j.cvsm.2007.03.003 17619007

[57] Benvenuti E Pierini A Bottero E Pietra M Gori E Salvadori S. Immunosuppressant-responsive enteropathy and non-responsive enteropathy in dogs: Prognostic factors, short- and long-term follow up. Animals. (2021) 11:2637. 10.3390/ani11092637 34573603 PMC8472317

[58] Mugnier A Mila H Guiraud F Brévaux J Lecarpentier M Martinez C. Birth weight as a risk factor for neonatal mortality: breed-specific approach to identify at-risk puppies. Prev Vet Med. (2019) 171:104746. 10.1016/j.prevetmed.2019.104746 31491708

[59] German AJ Ryan VH German AC Wood IS Trayhurn P. Obesity, its associated disorders and the role of inflammatory adipokines in companion animals. Vet J. (2010) 185:4–9. 10.1016/j.tvjl.2010.04.004 20472476

[60] Worthing KA Wigney DI Dhand NK Fawcett A McDonagh P Malik R. Risk factors for feline infectious peritonitis in Australian cats. J Feline Med Surg. (2012) 14:405–12. 10.1177/1098612X12441875 22398460 PMC10822597

[61] Prahl A Guptill L Glickman NW Tetrick M Glickman LT. Time trends and risk factors for diabetes mellitus in cats presented to veterinary teaching hospitals. J Feline Med Surg. (2007) 9:351–8. 10.1016/j.jfms.2007.02.004 17449313 PMC10832956

[62] Isomura R Yamazaki M Inoue M Kwan NC Matsuda M Sugiura K. The age, breed and sex pattern of diagnosis for veterinary care in insured cats in Japan. J Small Anim Pract. (2017) 58:89–95. 10.1111/jsap.12617 28160304

[63] Economu L Stell A O'Neill DG Schofield I Stevens K Brodbelt D. Incidence and risk factors for feline lymphoma in UK primary-care practice. J Small Anim Pract. (2021) 62:97–106. 10.1111/jsap.13266 33325082 PMC7986087

[64] Sparkes AH Caney S Chalhoub S Elliott J Finch N Gajanayake I. ISFM consensus guidelines on the diagnosis and management of feline chronic kidney disease. J Feline Med Surg. (2016) 18:219–39. 10.1177/1098612X16631234 26936494 PMC11148907

[65] Freeman LM Lachaud MP Matthews S Rhodes L Zollers B. Evaluation of weight loss over time in cats with chronic kidney disease. J Vet Intern Med. (2016) 30:1661–6. 10.1111/jvim.14561 27527534 PMC5032880

[66] Rowe E Browne W Casey R Gruffydd-Jones T Murray J. Risk factors identified for owner-reported feline obesity at around one year of age: dry diet and indoor lifestyle. Prev Vet Med. (2015) 121:273–81. 10.1016/j.prevetmed.2015.07.011 26265631

[67] Burkholder WJ. Use of body condition scores in clinical assessment of the provision of optimal nutrition. J Am Vet Med Assoc. (2000) 217:650–4. 10.2460/javma.2000.217.650 10976293

[68] Jeusette I Greco D Aquino F Detilleux J Peterson M Romano V. Effect of breed on body composition and comparison between various methods to estimate body composition in dogs. Res Vet Sci. (2010) 88:227–32. 10.1016/j.rvsc.2009.07.009 19692101

[69] Laflamme D. Development and validation of a body condition score system for dogs. Canine Pract. (1997) 22:10–5.

[70] Laflamme D. Development and validation of a body condition score system for cats: a clinical tool. Feline Pract. (1997) 25:13–8.

[71] Mawby DI Bartges JW d'Avignon A Laflamme DP Moyers TD Cottrell T. Comparison of various methods for estimating body fat in dogs. J Am Anim Hosp Assoc. (2004) 40:109–14. 10.5326/0400109 15007045

[72] Santarossa A Parr JM Verbrugghe A. The importance of assessing body composition of dogs and cats and methods available for use in clinical practice. J Am Vet Med Assoc. (2017) 251:521–9. 10.2460/javma.251.5.521 28828948

[73] Shoveller AK DiGennaro J Lanman C Spangler D. Trained vs untrained evaluator assessment of body condition score as a predictor of percent body fat in adult cats. J Feline Med Surg. (2014) 16:957–65. 10.1177/1098612X14527472 24626465 PMC11104095

[74] Skiadas CH Skiadas C. Direct healthy life expectancy estimates from life tables with a sullivan extension. Bridging the gap between hale and eurostat estimates. In:Skiadas CH Skiadas C, editors. Demography of Population Health, Aging and Health Expenditures. Cham: Springer International Publishing (2020). p. 25–42.

[75] Skiadas CH. Expanding the life tables for companion dogs in UK and Japan to include the healthy life expectancy. Version 1, last edited September 6, 2022. SocArXiv. (2022). 10.31235/osf.io/ftky9

