# Supplementary material for: Life expectancy tables for dogs and cats derived from clinical data
Source: Front Vet Sci. 2023 Feb 21;10:1082102. doi: 10.3389/fvets.2023.1082102 (PMC9989186; doi:10.3389/fvets.2023.1082102)
Supplement: Supplementary file 1 [file Data_Sheet_1.PDF]

**Supplement contents**

Supplementary Table 1. Total study populations (deceased and survivor) of dogs and cats by survey year ........ 2
Supplementary Table 2. Rates of return visits of dogs and cats by survey year ........ 3
Supplementary Table 3. Total study populations (deceased and survivor) of dogs and cats by sex and sterilization status ........ 4
Supplementary Table 4. Total study populations (deceased and survivor) of purebred and mixed-breed dogs and cats by age interval ........ 5
Supplementary Table 5. Total study populations (deceased and survivor) of purebred and mixed-breed dogs and cats by body condition score ........ 6
Supplementary Table 6. Total study populations (deceased and survivor) of dogs and cats by body condition score by year ........ 7
Supplementary Table 7. Life expectancies at birth of purebred and mixed-breed dogs and cats by survey year ........ 8
Supplementary Table 8. Life expectancies of purebred and mixed-breed dogs and cats by age interval and survey year ........ 9
Supplementary Table 9. Life expectancies of dogs and cats by age interval and sex ........ 14
Supplementary Table 10. Life expectancies of purebred and mixed-breed dogs and cats by age interval and sex ........ 15
Supplementary Table 11. Life expectancies of dogs by age interval and body condition score ........ 20
Supplementary Table 12. Life expectancies of cats by age interval and body condition score ........ 22
Supplementary Table 13. Life expectancies at birth of dogs and cats by survey year and body condition score ........ 24

**Supplementary Table 1. Total study populations (deceased and survivor) of dogs and cats by survey year**

| | Year of survey | | | | | | |
|---|---|---|---|---|---|---|---|
| | 2013 | 2014 | 2015 | 2016 | 2017 | 2018 | 2019 |
| **Dogs, n (%)** | | | | | | | |
| **All** | 1,625,465 | 1,746,393 | 1,838,930 | 1,922,181 | 1,996,815 | 2,061,890 | 2,101,255 |
| **Toy** | 343,931 (21.2) | 374,413 (21.4) | 398,655 (21.7) | 418,651 (21.8) | 434,370 (21.8) | 445,490 (21.6) | 448,831 (21.4) |
| **Small** | 486,809 (29.9) | 517,069 (29.6) | 537,641 (29.2) | 554,434 (28.8) | 568,162 (28.5) | 577,572 (28.0) | 580,165 (27.6) |
| **Medium** | 220,157 (13.5) | 233,013 (13.3) | 243,419 (13.2) | 253,306 (13.2) | 262,341 (13.1) | 271,937 (13.2) | 278,611 (13.3) |
| **Large** | 497,326 (30.6) | 537,997 (30.8) | 568,740 (30.9) | 600,060 (31.2) | 627,846 (31.4) | 650,409 (31.5) | 664,387 (31.6) |
| **Giant** | 22,500 (1.4) | 24,197 (1.4) | 25,831 (1.4) | 26,693 (1.4) | 27,494 (1.4) | 27,926 (1.4) | 28,046 (1.3) |
| **Mixed breed** | 54,742 (3.4) | 59,704 (3.4) | 64,644 (3.5) | 69,037 (3.6) | 76,602 (3.8) | 88,556 (4.3) | 101,215 (4.8) |
| **Cats, n (%)** | | | | | | | |
| **All** | 299,501 | 319,328 | 333,417 | 344,045 | 354,107 | 364,203 | 375,477 |
| **Purebred** | 48,221 (16.1) | 50,634 (15.9) | 51,880 (15.6) | 53,234 (15.5) | 55,635 (15.7) | 59,269 (16.3) | 62,339 (16.6) |
| **Mixed breed** | 251,280 (83.9) | 268,694 (84.1) | 281,537 (84.4) | 290,811 (84.5) | 298,472 (84.3) | 304,934 (83.7) | 313,138 (83.4) |

**Supplementary Table 2. Rates of return visits of dogs and cats by survey year**

| Year | Dogs | | | Cats | | |
|---|---|---|---|---|---|---|
| | Total population, n | Population returning within 2 years, n | Return rate, % | Total population, n | Population returning within 2 years, n | Return rate, % |
| **2013** | 1,625,465 | 1,215,470 | 74.8 | 299,501 | 184,076 | 61.5 |
| **2014** | 1,746,393 | 1,321,611 | 75.7 | 319,328 | 199,186 | 62.4 |
| **2015** | 1,838,930 | 1,406,273 | 76.5 | 333,417 | 212,093 | 63.6 |
| **2016** | 1,922,181 | 1,481,931 | 77.1 | 344,045 | 222,938 | 64.8 |
| **2017** | 1,996,815 | 1,549,539 | 77.6 | 354,107 | 231,714 | 65.4 |
| **2018** | 2,061,890 | 1,588,491 | 77.0 | 364,203 | 237,057 | 65.1 |
| **2019** | 2,101,255 | 1,594,157 | 75.9 | 375,477 | 234,642 | 62.5 |

**Supplementary Table 3. Total study populations (deceased and survivor) of dogs and cats by sex and sterilization status**

| | Female | | | Male | | |
|---|---|---|---|---|---|---|
| | Total | Spayed | Entire | Total | Neutered | Entire |
| **Dogs, n (%)** | | | | | | |
| **All** | 6,377,544 (48.0) | 5,824,584 (91.3) | 552,960 (8.7) | 6,915,385 (52.0) | 5,988,681 (86.6) | 926,704 (13.4) |
| **Toy** | 1,376,384 (48.1) | 1,201,899 (87.3) | 174,485 (12.7) | 1,487,957 (51.9) | 1,256,441 (84.4) | 231,516 (15.6) |
| **Small** | 1,770,300 (46.3) | 1,633,007 (92.2) | 137,293 (7.8) | 2,051,552 (53.7) | 1,823,934 (88.9) | 227,618 (11.1) |
| **Medium** | 861,999 (48.9) | 813,233 (94.3) | 48,766 (5.7) | 900,785 (51.1) | 812,791 (90.2) | 87,994 (9.8) |
| **Large** | 2,022,440 (48.8) | 1,858,113 (91.9) | 164,327 (8.1) | 2,124,325 (51.2) | 1,793,769 (84.4) | 330,556 (15.6) |
| **Giant** | 80,601 (44.1) | 71,265 (88.4) | 9,336 (11.6) | 102,086 (55.9) | 80,331 (78.7) | 21,755 (21.3) |
| **Mixed breed** | 265,820 (51.7) | 247,067 (92.9) | 18,753 (7.1) | 248,680 (48.3) | 221,415 (89.0) | 27,265 (11.0) |
| **Cats, n (%)** | | | | | | |
| **All** | 1,200,205 (50.2) | 1,153,123 (96.1) | 47,082 (3.9) | 1,189,873 (49.8) | 1,152,646 (96.9) | 37,227 (3.1) |
| **Purebred** | 206,314 (54.1) | 198,176 (96.1) | 8,138 (3.9) | 174,898 (45.9) | 169,515 (96.9) | 5,383 (3.1) |
| **Mixed breed** | 993,891 (49.5) | 954,947 (96.1) | 38,944 (3.9) | 1,014,975 (50.5) | 983,131 (96.9) | 31,844 (3.1) |

**Supplementary Table 4. Total study populations (deceased and survivor) of purebred and mixed-breed dogs and cats by age interval**

| | Dogs, n (%) | | | | | | Cats , n (%) | |
|---|---|---|---|---|---|---|---|---|
| Age interval, years | Purebred dog size group | | | | | Mixed-breed dogs | Purebred cats | Mixed-breed cats |
| | Toy | Small | Medium | Large | Giant | | | |
| **0–1** | 319,530 (19.3) | 344,599 (20.9) | 206,998 (12.5) | 673,838 (40.8) | 37,212 (2.3) | 70,301 (4.3) | 49,084 (14.3) | 295,311 (85.7) |
| **1–2** | 265,241 (19.7) | 308,981 (23.0) | 173,316 (12.9) | 512,931 (38.1) | 27,249 (2.0) | 58,476 (4.3) | 35,140 (14.7) | 203,859 (85.3) |
| **2–3** | 245,382 (20.6) | 291,429 (24.5) | 151,714 (12.8) | 428,259 (36.0) | 21,156 (1.8) | 50,962 (4.3) | 28,098 (14.7) | 163,018 (85.3) |
| **3–4** | 239,638 (21.7) | 289,106 (26.2) | 139,278 (12.6) | 373,711 (33.8) | 17,676 (1.6) | 45,407 (4.1) | 25,164 (15.0) | 142,399 (85.0) |
| **4–5** | 243,835 (22.6) | 299,695 (27.7) | 134,809 (12.5) | 343,215 (31.8) | 16,362 (1.5) | 42,223 (3.9) | 23,754 (15.3) | 131,496 (84.7) |
| **5–6** | 236,769 (23.1) | 299,264 (29.2) | 127,734 (12.5) | 307,597 (30.0) | 14,231 (1.4) | 38,588 (3.8) | 22,680 (15.7) | 121,619 (84.3) |
| **6–7** | 222,930 (23.3) | 291,443 (30.5) | 120,309 (12.6) | 273,446 (28.6) | 12,412 (1.3) | 34,314 (3.6) | 21,280 (16.3) | 109,376 (83.7) |
| **7–8** | 212,667 (23.3) | 289,927 (31.7) | 117,501 (12.9) | 251,771 (27.5) | 10,636 (1.2) | 31,665 (3.5) | 20,734 (16.6) | 104,524 (83.4) |
| **8–9** | 194,115 (23.0) | 275,681 (32.6) | 111,735 (13.2) | 226,828 (26.8) | 8,805 (1.0) | 28,517 (3.4) | 20,171 (16.8) | 100,236 (83.2) |
| **9–10** | 168,482 (22.4) | 251,001 (33.3) | 103,099 (13.7) | 198,889 (26.4) | 6,794 (0.9) | 24,945 (3.3) | 18,584 (17.0) | 90,431 (83.0) |
| **10–11** | 146,594 (21.9) | 227,209 (33.9) | 96,347 (14.4) | 173,009 (25.8) | 4,665 (0.7) | 22,833 (3.4) | 19,221 (17.3) | 91,910 (82.7) |
| **11–12** | 115,357 (21.2) | 189,525 (34.8) | 82,499 (15.1) | 136,731 (25.1) | 2,785 (0.5) | 18,459 (3.4) | 16,672 (17.4) | 78,906 (82.6) |
| **12–13** | 89,177 (20.5) | 155,410 (35.7) | 69,611 (16.0) | 104,520 (24.0) | 1,498 (0.3) | 15,705 (3.6) | 16,328 (17.5) | 77,038 (82.5) |
| **13–14** | 66,329 (20.3) | 121,224 (37.0) | 54,525 (16.7) | 71,898 (22.0) | 737 (0.2) | 12,533 (3.8) | 15,175 (17.8) | 70,080 (82.2) |
| **14–15** | 45,598 (20.7) | 86,624 (39.3) | 37,409 (17.0) | 41,154 (18.7) | 308 (0.1) | 9,059 (4.1) | 13,761 (18.0) | 62,873 (82.0) |
| **15–16** | 28,057 (21.9) | 54,059 (42.2) | 21,270 (16.6) | 18,746 (14.6) | 119 (0.1) | 5,714 (4.5) | 11,635 (17.5) | 55,012 (82.5) |
| **16–17** | 14,273 (23.1) | 28,307 (45.9) | 9,366 (15.2) | 6,895 (11.2) | 29 (0.0) | 2,834 (4.6) | 9,263 (18.0) | 42,121 (82.0) |
| **17 +** | 10,452 (26.3) | 18,435 (46.4) | 5,300 (13.3) | 3,452 (8.7) | 16 (0.0) | 2,056 (5.2) | 14,602 (17.0) | 71,205 (83.0) |

**Supplementary Table 5. Total study populations (deceased and survivor) of purebred and mixed-breed dogs and cats by body condition score**

| | Body condition score | | | | |
|---|---|---|---|---|---|
| | 1 | 2 | 3 | 4 | 5 |
| **Dogs, n (%)** | | | | | |
| **All** | 24,028 (0.2) | 179,048 (1.4) | 9,632,272 (72.7) | 3,210,236 (24.2) | 200,467 (1.5) |
| **Toy** | 4,651 (0.2) | 34,738 (1.2) | 2,198,462 (77.0) | 583,606 (20.4) | 33,520 (1.2) |
| **Small** | 5,460 (0.1) | 39,111 (1.0) | 2,759,686 (72.4) | 949,511 (24.9) | 55,518 (1.5) |
| **Medium** | 2,933 (0.2) | 23,628 (1.3) | 1,187,080 (67.6) | 505,932 (28.8) | 36,869 (2.1) |
| **Large** | 9,189 (0.2) | 69,206 (1.7) | 2,971,935 (71.9) | 1,015,140 (24.6) | 65,415 (1.6) |
| **Giant** | 419 (0.2) | 3,929 (2.2) | 144,363 (79.4) | 31,251 (17.2) | 1,785 (1.0) |
| **Mixed breed** | 1,376 (0.3) | 8,436 (1.6) | 370,746 (72.3) | 124,796 (24.3) | 7,360 (1.4) |
| **Cats, n (%)** | | | | | |
| **All** | 44,188 (1.9) | 187,188 (7.9) | 1,390,147 (58.5) | 671,025 (28.2) | 85,065 (3.6) |
| **Purebred** | 6,924 (1.8) | 32,017 (8.4) | 236,545 (62.4) | 92,637 (24.4) | 10,810 (2.9) |
| **Mixed breed** | 37,264 (1.9) | 155,171 (7.8) | 1,153,602 (57.7) | 578,388 (28.9) | 74,255 (3.7) |

**Supplementary Table 6. Total study populations (deceased and survivor) of dogs and cats by body condition score by year**

| Year | Body condition score | | | | |
|---|---|---|---|---|---|
| | 1 | 2 | 3 | 4 | 5 |
| **Dogs, n (%)** | | | | | |
| **2013** | 6,521 (0.4) | 40,065 (2.5) | 1,092,295 (68.7) | 418,524 (26.3) | 33,513 (2.1) |
| **2014** | 4,615 (0.3) | 33,532 (1.9) | 1,234,024 (70.8) | 438,926 (25.2) | 31,296 (1.8) |
| **2015** | 3,346 (0.2) | 27,091 (1.5) | 1,326,507 (72.2) | 450,862 (24.6) | 28,485 (1.6) |
| **2016** | 2,593 (0.1) | 22,498 (1.2) | 1,408,848 (73.4) | 459,614 (23.9) | 26,456 (1.4) |
| **2017** | 2,345 (0.1) | 20,197 (1.0) | 1,474,411 (73.9) | 472,356 (23.7) | 26,194 (1.3) |
| **2018** | 2,322 (0.1) | 18,471 (0.9) | 1,532,198 (74.3) | 481,162 (23.3) | 26,674 (1.3) |
| **2019** | 2,286 (0.1) | 17,194 (0.8) | 1,563,989 (74.5) | 488,792 (23.3) | 27,849 (1.3) |
| **Cats, n (%)** | | | | | |
| **2013** | 10,058 (3.5) | 32,012 (11.0) | 154,261 (53.2) | 81,316 (28.0) | 12,477 (4.3) |
| **2014** | 8,308 (2.6) | 31,441 (9.9) | 179,168 (56.3) | 87,122 (27.4) | 12,359 (3.9) |
| **2015** | 6,797 (2.0) | 29,170 (8.8) | 193,601 (58.2) | 91,471 (27.5) | 11,634 (3.5) |
| **2016** | 5,207 (1.5) | 26,001 (7.6) | 204,799 (59.6) | 95,837 (27.9) | 11,555 (3.4) |
| **2017** | 5,000 (1.4) | 24,499 (6.9) | 212,231 (60.0) | 100,373 (28.4) | 11,761 (3.3) |
| **2018** | 4,451 (1.2) | 22,367 (6.1) | 219,858 (60.4) | 105,135 (28.9) | 12,185 (3.3) |
| **2019** | 4,367 (1.2) | 21,698 (5.8) | 226,229 (60.3) | 109,771 (29.3) | 13,094 (3.5) |

Supplementary Table 7. Life expectancies at birth of purebred and mixed-breed dogs and cats by survey year

| | Year of survey | | | | | | | |
|---|---|---|---|---|---|---|---|---|
| | 2013 | 2014 | 2015 | 2016 | 2017 | 2018 | 2019 | Overall |
| **Dogs $LE_{birth}$, years (95% CI)** | | | | | | | | |
| **Purebred dogs by size group** | | | | | | | | |
| **Toy** | 12.85 (12.79–12.92) | 12.99 (12.93–13.06) | 13.22 (13.15–13.28) | 13.37 (13.32–13.43) | 13.53 (13.47–13.59) | 13.66 (13.6–13.71) | 13.58 (13.52–13.63) | 13.36 (13.33–13.38) |
| **Small** | 13.13 (13.08–13.18) | 13.19 (13.14–13.23) | 13.41 (13.36–13.45) | 13.58 (13.53–13.63) | 13.69 (13.64–13.73) | 13.79 (13.74–13.83) | 13.79 (13.74–13.83) | 13.53 (13.52–13.55) |
| **Medium** | 12.38 (12.32–12.44) | 12.52 (12.46–12.58) | 12.54 (12.49–12.6) | 12.75 (12.69–12.8) | 12.82 (12.76–12.87) | 12.87 (12.81–12.92) | 12.94 (12.88–12.99) | 12.7 (12.68–12.72) |
| **Large** | 11.22 (11.18–11.26) | 11.29 (11.25–11.33) | 11.39 (11.35–11.42) | 11.56 (11.53–11.6) | 11.6 (11.56–11.63) | 11.7 (11.66–11.73) | 11.7 (11.66–11.73) | 11.51 (11.49–11.52) |
| **Giant** | 9.1 (8.92–9.27) | 9.44 (9.27–9.61) | 9.48 (9.31–9.66) | 9.43 (9.27–9.58) | 9.53 (9.39–9.68) | 9.82 (9.63–10.02) | 9.7 (9.54–9.85) | 9.51 (9.45–9.58) |
| **Mixed breed** | 12.15 (12.02–12.28) | 12.4 (12.27–12.54) | 12.53 (12.4–12.65) | 12.66 (12.54–12.78) | 12.92 (12.8–13.03) | 12.98 (12.87–13.09) | 12.98 (12.88–13.08) | 12.71 (12.67–12.76) |
| **Cats $LE_{birth}$, years (95% CI)** | | | | | | | | |
| **Purebred** | 10.84 (10.68–11.0) | 11.03 (10.87–11.18) | 11.4 (11.25–11.56) | 11.65 (11.5–11.81) | 11.76 (11.61–11.91) | 12.1 (11.95–12.25) | 11.85 (11.7–12.0) | 11.54 (11.48–11.6) |
| **Mixed-breed** | 10.29 (10.22–10.35) | 10.44 (10.37–10.5) | 10.83 (10.76–10.89) | 11.24 (11.17–11.3) | 11.43 (11.37–11.49) | 11.79 (11.72–11.85) | 11.69 (11.63–11.76) | 11.12 (11.09–11.14) |

CI, confidence interval; $LE_{birth}$, life expectancy at birth.

**Supplementary Table 8. Life expectancies of purebred and mixed-breed dogs and cats by age interval and survey year**

| | Age interval, years | Year of survey | | | | | | |
|---|---|---|---|---|---|---|---|---|
| | | 2013 | 2014 | 2015 | 2016 | 2017 | 2018 | 2019 |
| **Dogs LE, years (95% CI)** | | | | | | | | |
| **Toy** | 0–1 | 12.85 (12.79–12.92) | 12.99 (12.93–13.06) | 13.22 (13.15–13.28) | 13.37 (13.32–13.43) | 13.53 (13.47–13.59) | 13.66 (13.6–13.71) | 13.58 (13.52–13.63) |
| | 1–2 | 12.36 (12.3–12.43) | 12.52 (12.45–12.58) | 12.66 (12.6–12.73) | 12.8 (12.75–12.86) | 12.94 (12.89–13.0) | 13.04 (12.99–13.1) | 12.97 (12.92–13.02) |
| | 2–3 | 11.52 (11.46–11.59) | 11.68 (11.61–11.74) | 11.81 (11.75–11.88) | 11.94 (11.89–12.0) | 12.08 (12.02–12.13) | 12.16 (12.11–12.21) | 12.09 (12.04–12.14) |
| | 3–4 | 10.65 (10.59–10.72) | 10.79 (10.73–10.85) | 10.94 (10.88–11.0) | 11.05 (11.0–11.11) | 11.19 (11.13–11.24) | 11.26 (11.21–11.31) | 11.19 (11.14–11.24) |
| | 4–5 | 9.77 (9.7–9.83) | 9.92 (9.86–9.98) | 10.04 (9.98–10.1) | 10.17 (10.11–10.22) | 10.28 (10.23–10.33) | 10.36 (10.31–10.41) | 10.28 (10.24–10.33) |
| | 5–6 | 8.88 (8.82–8.95) | 9.02 (8.96–9.08) | 9.15 (9.09–9.21) | 9.27 (9.21–9.32) | 9.38 (9.32–9.43) | 9.45 (9.4–9.5) | 9.38 (9.34–9.43) |
| | 6–7 | 8.01 (7.94–8.07) | 8.14 (8.08–8.2) | 8.28 (8.22–8.34) | 8.38 (8.33–8.44) | 8.49 (8.44–8.55) | 8.56 (8.51–8.61) | 8.49 (8.45–8.54) |
| | 7–8 | 7.15 (7.09–7.22) | 7.27 (7.21–7.34) | 7.41 (7.35–7.47) | 7.51 (7.46–7.57) | 7.62 (7.56–7.67) | 7.69 (7.64–7.73) | 7.62 (7.58–7.67) |
| | 8–9 | 6.32 (6.25–6.38) | 6.43 (6.37–6.5) | 6.56 (6.5–6.62) | 6.66 (6.6–6.71) | 6.77 (6.71–6.82) | 6.83 (6.78–6.88) | 6.77 (6.73–6.81) |
| | 9–10 | 5.53 (5.47–5.6) | 5.64 (5.58–5.7) | 5.77 (5.71–5.83) | 5.85 (5.8–5.91) | 5.96 (5.91–6.01) | 6.02 (5.97–6.07) | 5.97 (5.92–6.01) |
| | 10–11 | 4.81 (4.74–4.87) | 4.88 (4.82–4.95) | 5.04 (4.98–5.1) | 5.1 (5.04–5.15) | 5.2 (5.15–5.25) | 5.25 (5.21–5.3) | 5.22 (5.17–5.26) |
| | 11–12 | 4.17 (4.1–4.23) | 4.21 (4.14–4.27) | 4.35 (4.29–4.41) | 4.42 (4.37–4.48) | 4.5 (4.45–4.55) | 4.55 (4.5–4.6) | 4.52 (4.48–4.57) |
| | 12–13 | 3.55 (3.48–3.62) | 3.61 (3.54–3.67) | 3.76 (3.7–3.83) | 3.8 (3.74–3.86) | 3.87 (3.82–3.93) | 3.92 (3.87–3.97) | 3.88 (3.84–3.93) |
| | 13–14 | 3.05 (2.97–3.12) | 3.08 (3.01–3.15) | 3.23 (3.16–3.3) | 3.24 (3.18–3.3) | 3.32 (3.26–3.38) | 3.35 (3.3–3.41) | 3.32 (3.28–3.37) |
| | 14–15 | 2.59 (2.51–2.67) | 2.61 (2.54–2.69) | 2.77 (2.7–2.85) | 2.77 (2.71–2.84) | 2.84 (2.77–2.91) | 2.87 (2.81–2.93) | 2.85 (2.8–2.91) |
| | 15–16 | 2.26 (2.17–2.35) | 2.31 (2.22–2.4) | 2.44 (2.35–2.53) | 2.4 (2.32–2.48) | 2.47 (2.39–2.55) | 2.5 (2.43–2.57) | 2.47 (2.41–2.53) |
| | 16–17 | 2.04 (1.93–2.16) | 2.12 (2.0–2.23) | 2.21 (2.1–2.33) | 2.15 (2.06–2.25) | 2.26 (2.16–2.36) | 2.23 (2.14–2.31) | 2.23 (2.15–2.31) |
| | 17+ | 1.93 (1.77–2.09) | 1.92 (1.78–2.07) | 2.09 (1.93–2.24) | 1.99 (1.86–2.12) | 2.11 (1.98–2.24) | 2.07 (1.96–2.18) | 2.08 (1.97–2.18) |
| **Small** | 0–1 | 13.13 (13.08–13.18) | 13.19 (13.14–13.23) | 13.41 (13.36–13.45) | 13.58 (13.53–13.63) | 13.69 (13.64–13.73) | 13.79 (13.74–13.83) | 13.79 (13.74–13.83) |
| | 1–2 | 12.47 (12.42–12.52) | 12.51 (12.46–12.55) | 12.69 (12.64–12.73) | 12.84 (12.8–12.89) | 12.96 (12.92–13.0) | 13.04 (13.0–13.08) | 13.02 (12.98–13.06) |
| | 2–3 | 11.59 (11.55–11.64) | 11.64 (11.59–11.68) | 11.8 (11.76–11.84) | 11.94 (11.9–11.99) | 12.05 (12.01–12.09) | 12.13 (12.09–12.17) | 12.1 (12.07–12.14) |
| | 3–4 | 10.69 (10.64–10.73) | 10.73 (10.69–10.77) | 10.89 (10.85–10.93) | 11.02 (10.98–11.07) | 11.13 (11.09–11.17) | 11.2 (11.16–11.24) | 11.18 (11.14–11.21) |
| | 4–5 | 9.79 (9.74–9.83) | 9.84 (9.8–9.88) | 9.98 (9.94–10.02) | 10.12 (10.07–10.16) | 10.21 (10.17–10.25) | 10.28 (10.24–10.32) | 10.26 (10.22–10.3) |
| | 5–6 | 8.9 (8.85–8.94) | 8.94 (8.9–8.99) | 9.08 (9.04–9.12) | 9.21 (9.17–9.26) | 9.31 (9.27–9.35) | 9.36 (9.32–9.4) | 9.35 (9.31–9.39) |
| | 6–7 | 8.01 (7.97–8.05) | 8.06 (8.02–8.11) | 8.19 (8.15–8.23) | 8.31 (8.27–8.35) | 8.41 (8.37–8.45) | 8.46 (8.42–8.49) | 8.44 (8.41–8.48) |
| | 7–8 | 7.14 (7.1–7.18) | 7.19 (7.15–7.23) | 7.31 (7.27–7.35) | 7.43 (7.39–7.47) | 7.53 (7.5–7.57) | 7.57 (7.53–7.6) | 7.56 (7.52–7.59) |
| | 8–9 | 6.3 (6.26–6.35) | 6.34 (6.3–6.38) | 6.46 (6.42–6.5) | 6.58 (6.54–6.62) | 6.67 (6.63–6.71) | 6.7 (6.67–6.74) | 6.69 (6.65–6.72) |
| | 9–10 | 5.52 (5.47–5.56) | 5.54 (5.5–5.58) | 5.66 (5.62–5.7) | 5.76 (5.72–5.8) | 5.85 (5.82–5.89) | 5.89 (5.85–5.92) | 5.88 (5.85–5.91) |
| | 10–11 | 4.77 (4.72–4.81) | 4.79 (4.75–4.83) | 4.91 (4.87–4.95) | 5.01 (4.97–5.05) | 5.1 (5.06–5.13) | 5.12 (5.09–5.16) | 5.11 (5.08–5.15) |
| | 11–12 | 4.09 (4.05–4.14) | 4.11 (4.07–4.15) | 4.21 (4.18–4.25) | 4.32 (4.28–4.36) | 4.4 (4.36–4.44) | 4.42 (4.38–4.45) | 4.41 (4.37–4.44) |
| | 12–13 | 3.49 (3.44–3.53) | 3.51 (3.47–3.55) | 3.59 (3.55–3.63) | 3.68 (3.63–3.72) | 3.76 (3.72–3.8) | 3.78 (3.74–3.81) | 3.76 (3.73–3.79) |

| | | | | | | | | |
|---|---|---|---|---|---|---|---|---|
| | 13–14 | 2.96 (2.92–3.01) | 2.97 (2.92–3.01) | 3.04 (3.0–3.08) | 3.13 (3.09–3.17) | 3.2 (3.16–3.24) | 3.23 (3.19–3.26) | 3.2 (3.16–3.23) |
| | 14–15 | 2.52 (2.48–2.57) | 2.51 (2.47–2.56) | 2.6 (2.56–2.65) | 2.67 (2.62–2.71) | 2.74 (2.69–2.78) | 2.74 (2.7–2.79) | 2.73 (2.69–2.77) |
| | 15–16 | 2.19 (2.14–2.25) | 2.15 (2.1–2.2) | 2.28 (2.22–2.33) | 2.33 (2.28–2.38) | 2.39 (2.34–2.45) | 2.38 (2.33–2.42) | 2.36 (2.31–2.4) |
| | 16–17 | 1.96 (1.89–2.03) | 1.93 (1.87–2.0) | 2.02 (1.95–2.08) | 2.13 (2.06–2.2) | 2.17 (2.1–2.24) | 2.12 (2.06–2.18) | 2.1 (2.05–2.16) |
| | 17+ | 1.83 (1.73–1.93) | 1.79 (1.71–1.88) | 1.89 (1.79–1.98) | 1.99 (1.9–2.09) | 2.08 (1.99–2.18) | 2.01 (1.93–2.09) | 1.97 (1.89–2.04) |
| **Medium** | 0–1 | 12.38 (12.32–12.44) | 12.52 (12.46–12.58) | 12.54 (12.49–12.6) | 12.75 (12.69–12.8) | 12.82 (12.76–12.87) | 12.87 (12.81–12.92) | 12.94 (12.88–12.99) |
| | 1–2 | 11.68 (11.62–11.74) | 11.78 (11.72–11.83) | 11.77 (11.72–11.83) | 11.96 (11.9–12.02) | 12.03 (11.97–12.08) | 12.08 (12.02–12.13) | 12.14 (12.09–12.2) |
| | 2–3 | 10.78 (10.73–10.84) | 10.87 (10.82–10.93) | 10.88 (10.82–10.93) | 11.05 (11.0–11.11) | 11.12 (11.07–11.18) | 11.16 (11.1–11.21) | 11.23 (11.18–11.28) |
| | 3–4 | 9.88 (9.82–9.94) | 9.95 (9.9–10.01) | 9.96 (9.91–10.02) | 10.14 (10.08–10.19) | 10.2 (10.15–10.26) | 10.24 (10.18–10.29) | 10.31 (10.26–10.36) |
| | 4–5 | 8.98 (8.93–9.04) | 9.05 (9.0–9.11) | 9.04 (8.99–9.09) | 9.22 (9.17–9.28) | 9.29 (9.24–9.34) | 9.32 (9.27–9.37) | 9.38 (9.33–9.44) |
| | 5–6 | 8.1 (8.04–8.15) | 8.16 (8.11–8.22) | 8.14 (8.09–8.19) | 8.32 (8.27–8.37) | 8.37 (8.32–8.42) | 8.42 (8.37–8.47) | 8.49 (8.44–8.54) |
| | 6–7 | 7.23 (7.18–7.28) | 7.29 (7.23–7.34) | 7.27 (7.22–7.32) | 7.43 (7.38–7.48) | 7.47 (7.42–7.52) | 7.52 (7.47–7.57) | 7.58 (7.53–7.63) |
| | 7–8 | 6.37 (6.32–6.42) | 6.43 (6.38–6.48) | 6.4 (6.35–6.45) | 6.57 (6.52–6.62) | 6.61 (6.56–6.66) | 6.64 (6.6–6.69) | 6.71 (6.66–6.76) |
| | 8–9 | 5.59 (5.54–5.64) | 5.62 (5.57–5.66) | 5.58 (5.54–5.63) | 5.74 (5.7–5.79) | 5.77 (5.72–5.82) | 5.82 (5.77–5.86) | 5.87 (5.82–5.91) |
| | 9–10 | 4.82 (4.77–4.87) | 4.85 (4.8–4.9) | 4.81 (4.77–4.86) | 4.97 (4.92–5.02) | 5.0 (4.95–5.04) | 5.03 (4.99–5.08) | 5.09 (5.05–5.14) |
| | 10–11 | 4.12 (4.07–4.17) | 4.14 (4.09–4.19) | 4.1 (4.06–4.15) | 4.26 (4.21–4.31) | 4.28 (4.24–4.33) | 4.33 (4.29–4.37) | 4.36 (4.32–4.41) |
| | 11–12 | 3.51 (3.46–3.55) | 3.53 (3.48–3.57) | 3.49 (3.45–3.53) | 3.64 (3.59–3.68) | 3.65 (3.61–3.7) | 3.69 (3.65–3.74) | 3.73 (3.69–3.77) |
| | 12–13 | 2.97 (2.92–3.02) | 2.99 (2.94–3.03) | 2.96 (2.92–3.0) | 3.08 (3.04–3.13) | 3.12 (3.07–3.16) | 3.14 (3.09–3.18) | 3.16 (3.11–3.2) |
| | 13–14 | 2.51 (2.46–2.56) | 2.54 (2.49–2.58) | 2.5 (2.45–2.54) | 2.63 (2.58–2.68) | 2.66 (2.61–2.71) | 2.66 (2.62–2.71) | 2.69 (2.64–2.73) |
| | 14–15 | 2.18 (2.12–2.23) | 2.18 (2.12–2.23) | 2.14 (2.1–2.19) | 2.27 (2.21–2.33) | 2.33 (2.27–2.39) | 2.28 (2.23–2.33) | 2.32 (2.26–2.38) |
| | 15–16 | 1.92 (1.85–1.99) | 1.91 (1.84–1.98) | 1.87 (1.81–1.93) | 2.06 (1.98–2.13) | 2.1 (2.02–2.17) | 2.0 (1.94–2.06) | 2.06 (1.99–2.13) |
| | 16–17 | 1.8 (1.7–1.9) | 1.77 (1.68–1.87) | 1.7 (1.62–1.78) | 1.93 (1.82–2.04) | 1.94 (1.84–2.04) | 1.82 (1.73–1.9) | 1.9 (1.8–1.99) |
| | 17+ | 1.7 (1.56–1.83) | 1.66 (1.53–1.79) | 1.59 (1.48–1.71) | 1.82 (1.66–1.98) | 1.85 (1.7–1.99) | 1.76 (1.64–1.89) | 1.9 (1.75–2.05) |
| **Large** | 0–1 | 11.22 (11.18–11.26) | 11.29 (11.25–11.33) | 11.39 (11.35–11.42) | 11.56 (11.53–11.6) | 11.6 (11.56–11.63) | 11.7 (11.66–11.73) | 11.7 (11.66–11.73) |
| | 1–2 | 10.63 (10.59–10.67) | 10.69 (10.65–10.73) | 10.73 (10.7–10.77) | 10.88 (10.84–10.91) | 10.9 (10.86–10.93) | 10.98 (10.95–11.01) | 10.97 (10.93–11.0) |
| | 2–3 | 9.77 (9.74–9.81) | 9.83 (9.79–9.87) | 9.86 (9.83–9.9) | 10.0 (9.96–10.03) | 10.01 (9.98–10.04) | 10.09 (10.06–10.12) | 10.08 (10.05–10.12) |
| | 3–4 | 8.9 (8.87–8.94) | 8.94 (8.9–8.98) | 8.97 (8.94–9.01) | 9.09 (9.06–9.13) | 9.11 (9.08–9.14) | 9.19 (9.15–9.22) | 9.19 (9.15–9.22) |
| | 4–5 | 8.01 (7.98–8.05) | 8.06 (8.02–8.09) | 8.08 (8.04–8.11) | 8.2 (8.17–8.23) | 8.21 (8.18–8.24) | 8.28 (8.25–8.31) | 8.28 (8.25–8.31) |
| | 5–6 | 7.14 (7.1–7.18) | 7.18 (7.15–7.22) | 7.19 (7.16–7.23) | 7.31 (7.28–7.34) | 7.32 (7.29–7.35) | 7.39 (7.35–7.42) | 7.4 (7.37–7.43) |
| | 6–7 | 6.29 (6.26–6.33) | 6.33 (6.3–6.37) | 6.34 (6.31–6.37) | 6.45 (6.41–6.48) | 6.46 (6.43–6.49) | 6.52 (6.49–6.55) | 6.54 (6.51–6.57) |
| | 7–8 | 5.48 (5.45–5.52) | 5.52 (5.48–5.55) | 5.53 (5.5–5.57) | 5.63 (5.59–5.66) | 5.63 (5.6–5.66) | 5.69 (5.66–5.72) | 5.71 (5.68–5.74) |
| | 8–9 | 4.73 (4.7–4.77) | 4.76 (4.73–4.8) | 4.77 (4.74–4.81) | 4.86 (4.83–4.89) | 4.86 (4.83–4.89) | 4.91 (4.88–4.94) | 4.93 (4.9–4.96) |
| | 9–10 | 4.05 (4.02–4.09) | 4.09 (4.05–4.12) | 4.08 (4.05–4.11) | 4.16 (4.13–4.19) | 4.16 (4.13–4.19) | 4.21 (4.18–4.24) | 4.22 (4.19–4.25) |
| | 10–11 | 3.46 (3.43–3.49) | 3.49 (3.45–3.52) | 3.48 (3.45–3.51) | 3.54 (3.51–3.57) | 3.54 (3.51–3.57) | 3.59 (3.56–3.62) | 3.62 (3.59–3.65) |
| | 11–12 | 2.94 (2.91–2.98) | 2.98 (2.94–3.01) | 2.97 (2.94–3.0) | 3.03 (3.0–3.06) | 3.01 (2.98–3.04) | 3.05 (3.02–3.08) | 3.08 (3.05–3.11) |
| | 12–13 | 2.49 (2.46–2.53) | 2.52 (2.48–2.55) | 2.53 (2.5–2.56) | 2.58 (2.55–2.62) | 2.58 (2.54–2.61) | 2.59 (2.55–2.62) | 2.62 (2.58–2.65) |

| | | | | | | | | |
|---|---|---|---|---|---|---|---|---|
| | 13–14 | 2.14 (2.11–2.18) | 2.19 (2.15–2.23) | 2.19 (2.15–2.22) | 2.23 (2.19–2.26) | 2.23 (2.19–2.26) | 2.24 (2.21–2.28) | 2.25 (2.21–2.28) |
| | 14–15 | 1.89 (1.85–1.94) | 1.96 (1.91–2.01) | 1.92 (1.87–1.96) | 1.97 (1.92–2.01) | 1.97 (1.92–2.01) | 1.98 (1.93–2.02) | 1.98 (1.94–2.02) |
| | 15–16 | 1.73 (1.66–1.79) | 1.78 (1.71–1.84) | 1.75 (1.69–1.81) | 1.79 (1.73–1.86) | 1.79 (1.73–1.85) | 1.81 (1.74–1.87) | 1.79 (1.73–1.85) |
| | 16–17 | 1.58 (1.49–1.66) | 1.68 (1.57–1.78) | 1.61 (1.52–1.69) | 1.68 (1.58–1.77) | 1.66 (1.57–1.74) | 1.7 (1.6–1.79) | 1.66 (1.58–1.75) |
| | 17+ | 1.55 (1.41–1.68) | 1.63 (1.47–1.78) | 1.49 (1.36–1.62) | 1.6 (1.45–1.75) | 1.56 (1.43–1.68) | 1.6 (1.46–1.73) | 1.57 (1.44–1.69) |
| **Giant** | 0–1 | 9.1 (8.92–9.27) | 9.44 (9.27–9.61) | 9.48 (9.31–9.66) | 9.43 (9.27–9.58) | 9.53 (9.39–9.68) | 9.82 (9.63–10.02) | 9.7 (9.54–9.85) |
| | 1–2 | 8.38 (8.21–8.55) | 8.7 (8.53–8.87) | 8.77 (8.6–8.95) | 8.69 (8.54–8.85) | 8.77 (8.62–8.91) | 9.05 (8.85–9.24) | 8.93 (8.78–9.08) |
| | 2–3 | 7.55 (7.37–7.72) | 7.84 (7.67–8.0) | 7.94 (7.76–8.12) | 7.86 (7.71–8.02) | 7.93 (7.79–8.08) | 8.19 (8.0–8.39) | 8.08 (7.93–8.23) |
| | 3–4 | 6.71 (6.54–6.88) | 6.98 (6.82–7.15) | 7.1 (6.92–7.27) | 7.04 (6.89–7.19) | 7.11 (6.96–7.25) | 7.34 (7.14–7.53) | 7.21 (7.06–7.36) |
| | 4–5 | 5.9 (5.73–6.07) | 6.12 (5.96–6.29) | 6.26 (6.08–6.43) | 6.21 (6.06–6.36) | 6.27 (6.13–6.41) | 6.47 (6.27–6.66) | 6.37 (6.22–6.51) |
| | 5–6 | 5.1 (4.93–5.27) | 5.35 (5.18–5.52) | 5.51 (5.33–5.68) | 5.4 (5.26–5.55) | 5.46 (5.32–5.6) | 5.64 (5.44–5.84) | 5.59 (5.44–5.73) |
| | 6–7 | 4.36 (4.19–4.54) | 4.65 (4.48–4.82) | 4.8 (4.61–4.98) | 4.64 (4.49–4.79) | 4.75 (4.61–4.89) | 4.97 (4.77–5.17) | 4.87 (4.72–5.01) |
| | 7–8 | 3.76 (3.59–3.94) | 3.98 (3.81–4.15) | 4.19 (4.0–4.38) | 4.03 (3.88–4.18) | 4.1 (3.96–4.24) | 4.36 (4.15–4.57) | 4.21 (4.07–4.36) |
| | 8–9 | 3.26 (3.07–3.44) | 3.44 (3.26–3.62) | 3.63 (3.43–3.82) | 3.43 (3.28–3.58) | 3.55 (3.41–3.69) | 3.82 (3.59–4.05) | 3.64 (3.5–3.79) |
| | 9–10 | 2.92 (2.71–3.12) | 2.94 (2.75–3.12) | 3.15 (2.93–3.36) | 3.03 (2.86–3.2) | 3.04 (2.89–3.18) | 3.41 (3.16–3.67) | 3.18 (3.02–3.34) |
| | 10–11 | 2.63 (2.4–2.87) | 2.54 (2.33–2.74) | 2.78 (2.53–3.02) | 2.66 (2.47–2.84) | 2.67 (2.51–2.84) | 3.14 (2.83–3.44) | 2.82 (2.64–2.99) |
| | 11–12 | 2.34 (2.06–2.62) | 2.28 (2.03–2.54) | 2.5 (2.2–2.79) | 2.36 (2.14–2.58) | 2.3 (2.11–2.48) | 2.91 (2.54–3.29) | 2.52 (2.31–2.72) |
| | 12–13 | 2.16 (1.81–2.51) | 2.11 (1.78–2.43) | 2.37 (2.0–2.74) | 2.15 (1.87–2.42) | 2.08 (1.86–2.31) | 2.77 (2.29–3.26) | 2.32 (2.06–2.57) |
| | 13+ | 1.89 (1.44–2.34) | 2.09 (1.63–2.55) | 2.19 (1.71–2.67) | 1.95 (1.6–2.31) | 1.73 (1.46–2.0) | 2.73 (2.08–3.39) | 2.08 (1.75–2.41) |
| **Mixed-breed dogs** | 0–1 | 12.15 (12.02–12.28) | 12.4 (12.27–12.54) | 12.53 (12.4–12.65) | 12.66 (12.54–12.78) | 12.92 (12.8–13.03) | 12.98 (12.87–13.09) | 12.98 (12.88–13.08) |
| | 1–2 | 11.69 (11.56–11.82) | 11.95 (11.82–12.08) | 12.0 (11.88–12.11) | 12.04 (11.92–12.15) | 12.31 (12.2–12.42) | 12.29 (12.19–12.4) | 12.34 (12.24–12.43) |
| | 2–3 | 10.88 (10.75–11.01) | 11.11 (10.98–11.23) | 11.1 (10.98–11.21) | 11.19 (11.08–11.3) | 11.43 (11.32–11.54) | 11.39 (11.29–11.49) | 11.45 (11.35–11.54) |
| | 3–4 | 10.02 (9.89–10.14) | 10.22 (10.09–10.34) | 10.2 (10.09–10.32) | 10.29 (10.18–10.4) | 10.53 (10.42–10.63) | 10.51 (10.4–10.61) | 10.53 (10.44–10.62) |
| | 4–5 | 9.13 (9.01–9.26) | 9.32 (9.2–9.45) | 9.29 (9.18–9.4) | 9.41 (9.3–9.51) | 9.63 (9.53–9.74) | 9.61 (9.5–9.71) | 9.61 (9.52–9.7) |
| | 5–6 | 8.25 (8.13–8.37) | 8.41 (8.29–8.53) | 8.4 (8.29–8.51) | 8.51 (8.4–8.61) | 8.71 (8.61–8.81) | 8.67 (8.58–8.77) | 8.71 (8.62–8.79) |
| | 6–7 | 7.39 (7.27–7.51) | 7.5 (7.38–7.62) | 7.51 (7.4–7.61) | 7.64 (7.53–7.74) | 7.83 (7.72–7.93) | 7.81 (7.71–7.91) | 7.82 (7.73–7.9) |
| | 7–8 | 6.52 (6.4–6.64) | 6.64 (6.53–6.76) | 6.64 (6.54–6.75) | 6.76 (6.65–6.86) | 6.96 (6.86–7.06) | 6.92 (6.82–7.02) | 6.94 (6.85–7.02) |
| | 8–9 | 5.69 (5.58–5.81) | 5.85 (5.73–5.96) | 5.83 (5.72–5.93) | 5.94 (5.84–6.04) | 6.13 (6.03–6.23) | 6.11 (6.01–6.2) | 6.06 (5.98–6.15) |
| | 9–10 | 4.93 (4.82–5.04) | 5.08 (4.97–5.19) | 5.04 (4.94–5.14) | 5.16 (5.06–5.25) | 5.32 (5.22–5.42) | 5.35 (5.25–5.44) | 5.28 (5.2–5.36) |
| | 10–11 | 4.26 (4.15–4.37) | 4.4 (4.29–4.51) | 4.34 (4.24–4.44) | 4.43 (4.34–4.53) | 4.57 (4.48–4.67) | 4.63 (4.54–4.72) | 4.55 (4.47–4.63) |
| | 11–12 | 3.65 (3.54–3.76) | 3.75 (3.63–3.86) | 3.69 (3.59–3.79) | 3.79 (3.69–3.88) | 3.92 (3.83–4.02) | 3.95 (3.86–4.04) | 3.91 (3.83–3.99) |
| | 12–13 | 3.12 (3.01–3.23) | 3.17 (3.06–3.28) | 3.14 (3.04–3.24) | 3.24 (3.14–3.33) | 3.32 (3.22–3.41) | 3.32 (3.23–3.41) | 3.29 (3.21–3.37) |
| | 13–14 | 2.67 (2.55–2.78) | 2.76 (2.64–2.88) | 2.66 (2.56–2.77) | 2.72 (2.62–2.81) | 2.83 (2.73–2.93) | 2.82 (2.73–2.92) | 2.82 (2.73–2.9) |
| | 14–15 | 2.32 (2.2–2.44) | 2.43 (2.3–2.57) | 2.29 (2.18–2.4) | 2.3 (2.2–2.4) | 2.44 (2.33–2.55) | 2.46 (2.35–2.56) | 2.42 (2.33–2.52) |
| | 15–16 | 2.02 (1.87–2.16) | 2.15 (1.99–2.31) | 2.01 (1.88–2.14) | 2.03 (1.92–2.14) | 2.08 (1.95–2.2) | 2.18 (2.06–2.31) | 2.15 (2.04–2.26) |
| | 16–17 | 1.84 (1.65–2.03) | 2.02 (1.8–2.23) | 1.88 (1.7–2.06) | 1.8 (1.66–1.94) | 1.92 (1.76–2.08) | 1.96 (1.8–2.12) | 1.93 (1.79–2.07) |

| | | | | | | | | |
|---|---|---|---|---|---|---|---|---|
| | 17+ | 1.73 (1.47–1.99) | 1.89 (1.59–2.2) | 1.85 (1.59–2.11) | 1.56 (1.38–1.75) | 1.75 (1.53–1.97) | 1.85 (1.64–2.07) | 1.72 (1.53–1.9) |
| **Cats LE, years (95% CI)** | | | | | | | | |
| **Purebred cats** | 0–1 | 10.84 (10.69–11.0) | 11.03 (10.87–11.18) | 11.4 (11.25–11.56) | 11.65 (11.5–11.81) | 11.76 (11.61–11.91) | 12.1 (11.95–12.25) | 11.85 (11.71–12.0) |
| | 1–2 | 10.37 (10.22–10.52) | 10.58 (10.43–10.74) | 10.91 (10.76–11.06) | 11.12 (10.97–11.27) | 11.25 (11.1–11.4) | 11.55 (11.4–11.69) | 11.39 (11.25–11.53) |
| | 2–3 | 9.69 (9.54–9.84) | 9.9 (9.75–10.04) | 10.22 (10.07–10.37) | 10.42 (10.27–10.56) | 10.55 (10.41–10.69) | 10.8 (10.66–10.94) | 10.64 (10.5–10.78) |
| | 3–4 | 9.01 (8.87–9.15) | 9.17 (9.03–9.31) | 9.49 (9.35–9.63) | 9.65 (9.51–9.79) | 9.77 (9.64–9.91) | 10.08 (9.94–10.21) | 9.83 (9.7–9.97) |
| | 4–5 | 8.29 (8.15–8.43) | 8.44 (8.3–8.57) | 8.74 (8.6–8.87) | 8.88 (8.74–9.01) | 8.99 (8.86–9.12) | 9.27 (9.14–9.41) | 9.05 (8.92–9.18) |
| | 5–6 | 7.62 (7.49–7.75) | 7.7 (7.57–7.83) | 8.0 (7.88–8.13) | 8.12 (7.99–8.25) | 8.22 (8.09–8.35) | 8.46 (8.33–8.59) | 8.36 (8.24–8.49) |
| | 6–7 | 6.92 (6.8–7.05) | 6.98 (6.86–7.11) | 7.28 (7.15–7.4) | 7.43 (7.3–7.55) | 7.49 (7.37–7.61) | 7.75 (7.62–7.87) | 7.61 (7.49–7.73) |
| | 7–8 | 6.23 (6.12–6.35) | 6.29 (6.17–6.41) | 6.57 (6.45–6.69) | 6.76 (6.64–6.87) | 6.76 (6.64–6.88) | 7.0 (6.88–7.12) | 6.89 (6.77–7.0) |
| | 8–9 | 5.58 (5.47–5.7) | 5.63 (5.52–5.74) | 5.92 (5.81–6.04) | 6.11 (6.0–6.22) | 6.05 (5.94–6.16) | 6.31 (6.2–6.42) | 6.21 (6.11–6.32) |
| | 9–10 | 4.99 (4.89–5.1) | 4.98 (4.88–5.09) | 5.33 (5.22–5.44) | 5.48 (5.37–5.58) | 5.42 (5.31–5.52) | 5.63 (5.52–5.73) | 5.57 (5.47–5.67) |
| | 10–11 | 4.38 (4.28–4.48) | 4.46 (4.36–4.56) | 4.67 (4.57–4.77) | 4.8 (4.7–4.9) | 4.81 (4.71–4.91) | 4.96 (4.86–5.06) | 4.91 (4.81–5.01) |
| | 11–12 | 3.88 (3.78–3.98) | 4.03 (3.93–4.12) | 4.2 (4.1–4.3) | 4.26 (4.16–4.35) | 4.27 (4.17–4.36) | 4.45 (4.35–4.54) | 4.38 (4.29–4.48) |
| | 12–13 | 3.43 (3.34–3.52) | 3.53 (3.44–3.62) | 3.69 (3.6–3.78) | 3.76 (3.67–3.85) | 3.71 (3.62–3.8) | 3.93 (3.83–4.02) | 3.84 (3.76–3.93) |
| | 13–14 | 3.05 (2.96–3.14) | 3.11 (3.02–3.19) | 3.22 (3.13–3.3) | 3.29 (3.2–3.38) | 3.31 (3.22–3.39) | 3.43 (3.34–3.52) | 3.35 (3.27–3.44) |
| | 14–15 | 2.72 (2.63–2.8) | 2.75 (2.67–2.84) | 2.81 (2.72–2.89) | 2.91 (2.82–2.99) | 2.93 (2.85–3.02) | 3.03 (2.94–3.12) | 2.92 (2.84–3.01) |
| | 15–16 | 2.39 (2.3–2.47) | 2.45 (2.36–2.53) | 2.54 (2.46–2.63) | 2.51 (2.42–2.59) | 2.63 (2.54–2.72) | 2.66 (2.57–2.75) | 2.61 (2.53–2.7) |
| | 16–17 | 2.13 (2.04–2.22) | 2.19 (2.09–2.28) | 2.23 (2.14–2.33) | 2.22 (2.13–2.31) | 2.34 (2.25–2.44) | 2.37 (2.27–2.46) | 2.35 (2.25–2.44) |
| | 17+ | 1.93 (1.82–2.03) | 2.0 (1.89–2.11) | 2.04 (1.92–2.15) | 2.0 (1.89–2.1) | 2.13 (2.02–2.24) | 2.1 (1.99–2.22) | 2.14 (2.03–2.26) |
| **Mixed-breed cats** | 0–1 | 10.29 (10.22–10.35) | 10.44 (10.37–10.5) | 10.83 (10.76–10.89) | 11.24 (11.17–11.3) | 11.43 (11.37–11.49) | 11.79 (11.72–11.85) | 11.69 (11.63–11.76) |
| | 1–2 | 9.97 (9.91–10.04) | 10.13 (10.07–10.2) | 10.45 (10.39–10.52) | 10.82 (10.76–10.88) | 11.0 (10.94–11.07) | 11.32 (11.26–11.39) | 11.24 (11.18–11.31) |
| | 2–3 | 9.36 (9.3–9.43) | 9.48 (9.42–9.55) | 9.8 (9.74–9.87) | 10.15 (10.08–10.21) | 10.34 (10.28–10.4) | 10.63 (10.57–10.69) | 10.55 (10.49–10.61) |
| | 3–4 | 8.71 (8.65–8.77) | 8.82 (8.76–8.88) | 9.1 (9.04–9.16) | 9.43 (9.37–9.49) | 9.62 (9.56–9.68) | 9.88 (9.82–9.94) | 9.81 (9.75–9.87) |
| | 4–5 | 8.04 (7.98–8.1) | 8.14 (8.08–8.2) | 8.4 (8.34–8.45) | 8.69 (8.63–8.75) | 8.88 (8.82–8.94) | 9.11 (9.05–9.17) | 9.06 (9.0–9.11) |
| | 5–6 | 7.35 (7.29–7.4) | 7.44 (7.38–7.5) | 7.69 (7.63–7.75) | 7.95 (7.89–8.01) | 8.12 (8.07–8.18) | 8.34 (8.28–8.39) | 8.3 (8.24–8.35) |
| | 6–7 | 6.68 (6.63–6.74) | 6.79 (6.74–6.85) | 7.0 (6.94–7.05) | 7.26 (7.2–7.31) | 7.39 (7.34–7.44) | 7.6 (7.54–7.65) | 7.56 (7.51–7.62) |
| | 7–8 | 6.01 (5.96–6.07) | 6.14 (6.08–6.19) | 6.29 (6.24–6.35) | 6.55 (6.5–6.6) | 6.67 (6.62–6.72) | 6.86 (6.81–6.91) | 6.83 (6.78–6.88) |
| | 8–9 | 5.38 (5.33–5.43) | 5.5 (5.45–5.55) | 5.65 (5.6–5.7) | 5.86 (5.81–5.9) | 5.97 (5.93–6.02) | 6.15 (6.1–6.2) | 6.13 (6.08–6.18) |
| | 9–10 | 4.81 (4.76–4.85) | 4.92 (4.87–4.97) | 5.05 (5.01–5.1) | 5.23 (5.19–5.28) | 5.32 (5.28–5.37) | 5.49 (5.45–5.54) | 5.5 (5.45–5.54) |
| | 10–11 | 4.22 (4.18–4.27) | 4.36 (4.31–4.4) | 4.47 (4.42–4.51) | 4.63 (4.59–4.68) | 4.71 (4.67–4.75) | 4.86 (4.81–4.9) | 4.87 (4.82–4.91) |
| | 11–12 | 3.78 (3.74–3.82) | 3.89 (3.85–3.93) | 3.98 (3.93–4.02) | 4.14 (4.09–4.18) | 4.18 (4.14–4.22) | 4.32 (4.28–4.36) | 4.34 (4.3–4.38) |
| | 12–13 | 3.32 (3.28–3.36) | 3.39 (3.35–3.43) | 3.48 (3.44–3.52) | 3.63 (3.59–3.67) | 3.65 (3.61–3.69) | 3.78 (3.74–3.82) | 3.8 (3.76–3.84) |
| | 13–14 | 2.94 (2.9–2.98) | 2.98 (2.94–3.02) | 3.08 (3.04–3.12) | 3.18 (3.14–3.21) | 3.22 (3.18–3.26) | 3.35 (3.31–3.39) | 3.34 (3.3–3.38) |
| | 14–15 | 2.6 (2.56–2.63) | 2.62 (2.59–2.66) | 2.71 (2.68–2.75) | 2.77 (2.74–2.81) | 2.81 (2.77–2.85) | 2.96 (2.92–3.0) | 2.94 (2.9–2.98) |
| | 15–16 | 2.29 (2.25–2.32) | 2.32 (2.28–2.36) | 2.38 (2.35–2.42) | 2.46 (2.42–2.49) | 2.47 (2.43–2.51) | 2.62 (2.57–2.66) | 2.61 (2.57–2.65) |

| | | | | | | | | |
|---|---|---|---|---|---|---|---|---|
| | 16–17 | 2.07 (2.03–2.11) | 2.07 (2.03–2.1) | 2.12 (2.08–2.16) | 2.21 (2.17–2.25) | 2.21 (2.17–2.25) | 2.32 (2.28–2.37) | 2.34 (2.3–2.38) |
| | 17+ | 1.87 (1.82–1.91) | 1.88 (1.83–1.92) | 1.91 (1.87–1.96) | 2.0 (1.95–2.04) | 1.98 (1.93–2.02) | 2.1 (2.05–2.15) | 2.09 (2.04–2.14) |

Dogs by size group are purebred dogs only.
CI, confidence interval; LE, life expectancy.

**Supplementary Table 9. Life expectancies of dogs and cats by age interval and sex**

| Age interval, years | Life expectancy, years (95% confidence interval) | | | |
|---|---|---|---|---|
| | Dogs | | Cats | |
| | Female | Male | Female | Male |
| 0–1 | 12.76 (12.75–12.77) | 12.63 (12.62–12.64) | 11.68 (11.65–11.71) | 10.72 (10.68–10.75) |
| 1–2 | 12.09 (12.08–12.11) | 11.98 (11.97–11.99) | 11.25 (11.22–11.29) | 10.33 (10.3–10.36) |
| 2–3 | 11.21 (11.2–11.22) | 11.11 (11.1–11.12) | 10.54 (10.51–10.57) | 9.7 (9.67–9.73) |
| 3–4 | 10.3 (10.29–10.31) | 10.22 (10.21–10.24) | 9.76 (9.73–9.79) | 9.04 (9.01–9.07) |
| 4–5 | 9.4 (9.39–9.41) | 9.33 (9.32–9.35) | 8.98 (8.95–9.01) | 8.36 (8.34–8.39) |
| 5–6 | 8.5 (8.49–8.51) | 8.45 (8.44–8.46) | 8.2 (8.17–8.23) | 7.67 (7.64–7.7) |
| 6–7 | 7.62 (7.61–7.64) | 7.58 (7.57–7.6) | 7.45 (7.42–7.48) | 7.01 (6.98–7.04) |
| 7–8 | 6.77 (6.76–6.78) | 6.74 (6.73–6.75) | 6.71 (6.68–6.74) | 6.34 (6.31–6.36) |
| 8–9 | 5.95 (5.94–5.96) | 5.94 (5.92–5.95) | 6.01 (5.99–6.04) | 5.69 (5.66–5.71) |
| 9–10 | 5.19 (5.18–5.2) | 5.19 (5.18–5.2) | 5.37 (5.34–5.39) | 5.09 (5.07–5.12) |
| 10–11 | 4.49 (4.48–4.5) | 4.51 (4.5–4.52) | 4.74 (4.72–4.76) | 4.5 (4.48–4.52) |
| 11–12 | 3.86 (3.85–3.87) | 3.9 (3.89–3.91) | 4.21 (4.19–4.23) | 4.03 (4.01–4.05) |
| 12–13 | 3.3 (3.29–3.31) | 3.36 (3.35–3.37) | 3.68 (3.66–3.69) | 3.54 (3.52–3.56) |
| 13–14 | 2.84 (2.82–2.85) | 2.9 (2.89–2.91) | 3.23 (3.21–3.25) | 3.12 (3.1–3.14) |
| 14–15 | 2.46 (2.44–2.47) | 2.53 (2.51–2.54) | 2.83 (2.82–2.85) | 2.75 (2.73–2.77) |
| 15–16 | 2.18 (2.16–2.19) | 2.24 (2.22–2.26) | 2.5 (2.49–2.52) | 2.43 (2.41–2.45) |
| 16–17 | 1.99 (1.97–2.01) | 2.04 (2.01–2.06) | 2.23 (2.21–2.24) | 2.19 (2.16–2.21) |
| 17 + | 1.89 (1.86–1.92) | 1.91 (1.88–1.94) | 2.01 (1.99–2.03) | 1.97 (1.94–1.99) |

**Supplementary Table 10. Life expectancies of purebred and mixed-breed dogs and cats by age interval and sex**

| | Age interval, years | Sex | |
|---|---|---|---|
| | | Female | Male |
| **Dogs LE, years (95% CI)** | | | |
| **Toy** | 0–1 | 13.32 (13.28–13.35) | 13.39 (13.36–13.42) |
| | 1–2 | 12.76 (12.73–12.79) | 12.85 (12.82–12.88) |
| | 2–3 | 11.9 (11.87–11.93) | 12.0 (11.97–12.03) |
| | 3–4 | 11.0 (10.97–11.03) | 11.12 (11.09–11.15) |
| | 4–5 | 10.1 (10.08–10.13) | 10.23 (10.2–10.26) |
| | 5–6 | 9.21 (9.18–9.24) | 9.34 (9.31–9.37) |
| | 6–7 | 8.33 (8.3–8.36) | 8.46 (8.43–8.48) |
| | 7–8 | 7.46 (7.43–7.49) | 7.58 (7.56–7.61) |
| | 8–9 | 6.61 (6.58–6.64) | 6.73 (6.7–6.76) |
| | 9–10 | 5.82 (5.79–5.85) | 5.93 (5.9–5.96) |
| | 10–11 | 5.07 (5.04–5.1) | 5.18 (5.15–5.2) |
| | 11–12 | 4.38 (4.35–4.41) | 4.49 (4.46–4.52) |
| | 12–13 | 3.76 (3.73–3.79) | 3.86 (3.83–3.89) |
| | 13–14 | 3.22 (3.19–3.25) | 3.3 (3.27–3.33) |
| | 14–15 | 2.76 (2.72–2.79) | 2.82 (2.79–2.86) |
| | 15–16 | 2.42 (2.38–2.46) | 2.44 (2.4–2.48) |
| | 16–17 | 2.21 (2.16–2.26) | 2.18 (2.13–2.23) |
| | 17+ | 2.07 (2.0–2.14) | 2.02 (1.95–2.09) |
| **Small** | 0–1 | 13.55 (13.52–13.57) | 13.52 (13.5–13.55) |
| | 1–2 | 12.82 (12.8–12.85) | 12.82 (12.79–12.84) |
| | 2–3 | 11.93 (11.9–11.95) | 11.92 (11.9–11.94) |
| | 3–4 | 11.0 (10.98–11.03) | 11.01 (10.99–11.03) |
| | 4–5 | 10.09 (10.07–10.11) | 10.11 (10.09–10.13) |
| | 5–6 | 9.19 (9.17–9.21) | 9.21 (9.19–9.23) |
| | 6–7 | 8.3 (8.27–8.32) | 8.31 (8.29–8.33) |
| | 7–8 | 7.42 (7.4–7.44) | 7.43 (7.41–7.45) |
| | 8–9 | 6.56 (6.54–6.58) | 6.58 (6.56–6.6) |

| | | | |
|---|---|---|---|
| | 9–10 | 5.76 (5.74–5.78) | 5.77 (5.75–5.79) |
| | 10–11 | 5.0 (4.98–5.02) | 5.02 (5.0–5.04) |
| | 11–12 | 4.3 (4.28–4.32) | 4.33 (4.31–4.35) |
| | 12–13 | 3.66 (3.64–3.68) | 3.7 (3.68–3.72) |
| | 13–14 | 3.11 (3.09–3.13) | 3.15 (3.12–3.17) |
| | 14–15 | 2.65 (2.62–2.67) | 2.69 (2.66–2.71) |
| | 15–16 | 2.29 (2.27–2.32) | 2.33 (2.31–2.36) |
| | 16–17 | 2.06 (2.02–2.09) | 2.1 (2.06–2.13) |
| | 17+ | 1.95 (1.9–2.0) | 1.96 (1.91–2.0) |
| **Medium** | 0–1 | 12.8 (12.77–12.83) | 12.6 (12.57–12.63) |
| | 1–2 | 12.04 (12.01–12.07) | 11.83 (11.8–11.86) |
| | 2–3 | 11.12 (11.09–11.15) | 10.93 (10.9–10.96) |
| | 3–4 | 10.19 (10.17–10.22) | 10.03 (10.0–10.05) |
| | 4–5 | 9.27 (9.25–9.3) | 9.12 (9.09–9.15) |
| | 5–6 | 8.36 (8.34–8.39) | 8.23 (8.2–8.26) |
| | 6–7 | 7.47 (7.45–7.5) | 7.35 (7.32–7.37) |
| | 7–8 | 6.6 (6.58–6.63) | 6.48 (6.46–6.51) |
| | 8–9 | 5.78 (5.75–5.8) | 5.67 (5.64–5.69) |
| | 9–10 | 5.0 (4.98–5.03) | 4.9 (4.87–4.92) |
| | 10–11 | 4.28 (4.26–4.3) | 4.2 (4.17–4.22) |
| | 11–12 | 3.65 (3.63–3.67) | 3.58 (3.56–3.6) |
| | 12–13 | 3.09 (3.06–3.11) | 3.05 (3.02–3.07) |
| | 13–14 | 2.61 (2.59–2.64) | 2.6 (2.57–2.62) |
| | 14–15 | 2.24 (2.22–2.27) | 2.25 (2.22–2.29) |
| | 15–16 | 1.97 (1.94–2.0) | 2.02 (1.98–2.06) |
| | 16–17 | 1.81 (1.77–1.86) | 1.87 (1.81–1.93) |
| | 17+ | 1.73 (1.67–1.8) | 1.79 (1.71–1.87) |
| **Large** | 0–1 | 11.74 (11.72–11.76) | 11.28 (11.26–11.3) |
| | 1–2 | 11.06 (11.04–11.08) | 10.62 (10.6–10.64) |
| | 2–3 | 10.18 (10.16–10.2) | 9.75 (9.73–9.77) |
| | 3–4 | 9.27 (9.25–9.29) | 8.87 (8.85–8.89) |
| | 4–5 | 8.37 (8.35–8.38) | 7.98 (7.96–8.0) |
| | 5–6 | 7.47 (7.45–7.49) | 7.1 (7.09–7.12) |

| | | | |
|---|---|---|---|
| | 6–7 | 6.6 (6.59–6.62) | 6.26 (6.24–6.27) |
| | 7–8 | 5.77 (5.75–5.79) | 5.44 (5.43–5.46) |
| | 8–9 | 4.99 (4.97–5.0) | 4.69 (4.68–4.71) |
| | 9–10 | 4.27 (4.26–4.29) | 4.01 (4.0–4.03) |
| | 10–11 | 3.64 (3.62–3.66) | 3.42 (3.41–3.44) |
| | 11–12 | 3.1 (3.09–3.12) | 2.91 (2.9–2.93) |
| | 12–13 | 2.63 (2.61–2.65) | 2.48 (2.46–2.5) |
| | 13–14 | 2.27 (2.25–2.29) | 2.14 (2.12–2.16) |
| | 14–15 | 1.99 (1.97–2.01) | 1.91 (1.88–1.93) |
| | 15–16 | 1.8 (1.77–1.83) | 1.74 (1.71–1.78) |
| | 16–17 | 1.67 (1.62–1.71) | 1.63 (1.58–1.68) |
| | 17+ | 1.58 (1.52–1.65) | 1.55 (1.47–1.62) |
| **Giant** | 0–1 | 9.76 (9.66–9.85) | 9.33 (9.24–9.41) |
| | 1–2 | 9.01 (8.91–9.1) | 8.59 (8.51–8.67) |
| | 2–3 | 8.13 (8.04–8.22) | 7.77 (7.69–7.85) |
| | 3–4 | 7.27 (7.18–7.36) | 6.94 (6.86–7.02) |
| | 4–5 | 6.41 (6.32–6.5) | 6.11 (6.03–6.19) |
| | 5–6 | 5.59 (5.5–5.68) | 5.34 (5.26–5.42) |
| | 6–7 | 4.86 (4.77–4.95) | 4.64 (4.56–4.72) |
| | 7–8 | 4.2 (4.11–4.29) | 4.03 (3.95–4.11) |
| | 8–9 | 3.62 (3.52–3.71) | 3.5 (3.41–3.58) |
| | 9–10 | 3.12 (3.02–3.22) | 3.09 (2.99–3.18) |
| | 10–11 | 2.73 (2.62–2.84) | 2.77 (2.67–2.88) |
| | 11–12 | 2.41 (2.28–2.54) | 2.5 (2.38–2.63) |
| | 12–13 | 2.23 (2.06–2.4) | 2.31 (2.15–2.46) |
| | 13+ | 2.03 (1.8–2.26) | 2.11 (1.91–2.3) |
| **Mixed-breed dogs** | 0–1 | 12.81 (12.75–12.87) | 12.61 (12.54–12.67) |
| | 1–2 | 12.2 (12.14–12.25) | 12.05 (11.99–12.11) |
| | 2–3 | 11.32 (11.26–11.37) | 11.19 (11.13–11.25) |
| | 3–4 | 10.41 (10.36–10.47) | 10.3 (10.24–10.36) |
| | 4–5 | 9.5 (9.45–9.56) | 9.42 (9.36–9.48) |
| | 5–6 | 8.6 (8.54–8.65) | 8.51 (8.45–8.57) |
| | 6–7 | 7.71 (7.66–7.76) | 7.63 (7.57–7.69) |
| | 7–8 | 6.83 (6.78–6.88) | 6.76 (6.7–6.82) |

| | | | |
|---|---|---|---|
| | 8–9 | 6.0 (5.95–6.05) | 5.93 (5.88–5.99) |
| | 9–10 | 5.22 (5.17–5.27) | 5.15 (5.1–5.21) |
| | 10–11 | 4.5 (4.45–4.55) | 4.45 (4.4–4.51) |
| | 11–12 | 3.85 (3.81–3.9) | 3.8 (3.74–3.86) |
| | 12–13 | 3.25 (3.21–3.3) | 3.23 (3.17–3.28) |
| | 13–14 | 2.77 (2.72–2.82) | 2.76 (2.7–2.82) |
| | 14–15 | 2.39 (2.34–2.44) | 2.39 (2.32–2.45) |
| | 15–16 | 2.1 (2.04–2.17) | 2.08 (2.01–2.16) |
| | 16–17 | 1.92 (1.84–2.0) | 1.9 (1.8–2.0) |
| | 17+ | 1.76 (1.65–1.86) | 1.77 (1.63–1.91) |
| **Cats LE, years (CI 95%)** | | | |
| **Purebred cats** | 0–1 | 11.98 (11.91–12.06) | 11.05 (10.97–11.14) |
| | 1–2 | 11.47 (11.39–11.54) | 10.58 (10.5–10.66) |
| | 2–3 | 10.72 (10.65–10.8) | 9.9 (9.82–9.98) |
| | 3–4 | 9.93 (9.86–10.0) | 9.21 (9.13–9.28) |
| | 4–5 | 9.12 (9.05–9.19) | 8.49 (8.42–8.57) |
| | 5–6 | 8.34 (8.28–8.41) | 7.79 (7.72–7.86) |
| | 6–7 | 7.59 (7.53–7.65) | 7.11 (7.04–7.18) |
| | 7–8 | 6.85 (6.79–6.91) | 6.44 (6.37–6.5) |
| | 8–9 | 6.15 (6.09–6.2) | 5.8 (5.74–5.87) |
| | 9–10 | 5.49 (5.44–5.54) | 5.2 (5.14–5.26) |
| | 10–11 | 4.82 (4.77–4.87) | 4.61 (4.55–4.67) |
| | 11–12 | 4.3 (4.25–4.35) | 4.13 (4.07–4.18) |
| | 12–13 | 3.77 (3.72–3.81) | 3.64 (3.58–3.69) |
| | 13–14 | 3.31 (3.26–3.35) | 3.19 (3.14–3.24) |
| | 14–15 | 2.91 (2.86–2.95) | 2.83 (2.78–2.88) |
| | 15–16 | 2.58 (2.54–2.62) | 2.51 (2.46–2.56) |
| | 16–17 | 2.27 (2.23–2.32) | 2.26 (2.2–2.32) |
| | 17+ | 2.06 (2.0–2.11) | 2.04 (1.98–2.11) |
| **Mixed-breed cats** | 0–1 | 11.62 (11.58–11.65) | 10.66 (10.63–10.7) |
| | 1–2 | 11.21 (11.17–11.24) | 10.29 (10.26–10.32) |
| | 2–3 | 10.5 (10.46–10.53) | 9.67 (9.63–9.7) |
| | 3–4 | 9.73 (9.7–9.76) | 9.01 (8.98–9.05) |

| | | | |
|---|---|---|---|
| | 4–5 | 8.95 (8.92–8.98) | 8.34 (8.31–8.37) |
| | 5–6 | 8.17 (8.14–8.2) | 7.65 (7.62–7.68) |
| | 6–7 | 7.42 (7.39–7.45) | 6.99 (6.96–7.02) |
| | 7–8 | 6.68 (6.65–6.71) | 6.32 (6.29–6.35) |
| | 8–9 | 5.98 (5.96–6.01) | 5.67 (5.64–5.69) |
| | 9–10 | 5.34 (5.31–5.36) | 5.07 (5.05–5.1) |
| | 10–11 | 4.72 (4.7–4.75) | 4.48 (4.46–4.51) |
| | 11–12 | 4.19 (4.17–4.21) | 4.01 (3.99–4.03) |
| | 12–13 | 3.66 (3.63–3.68) | 3.52 (3.5–3.54) |
| | 13–14 | 3.22 (3.2–3.24) | 3.11 (3.09–3.13) |
| | 14–15 | 2.82 (2.8–2.84) | 2.74 (2.72–2.76) |
| | 15–16 | 2.49 (2.47–2.51) | 2.42 (2.4–2.44) |
| | 16–17 | 2.21 (2.19–2.24) | 2.17 (2.15–2.2) |
| | 17+ | 2.0 (1.97–2.02) | 1.95 (1.92–1.98) |

Dogs by size group are purebred dogs only.
CI, confidence interval; LE, life expectancy.

**Supplementary Table 11. Life expectancies of dogs by age interval and body condition score**

| Age interval, years | BCS 1 | | | BCS 2 | | | BCS 3 | | | BCS 4 | | | BCS 5 | | |
|---|---|---|---|---|---|---|---|---|---|---|---|---|---|---|---|
| | P(x) | d(x) | LE, years (95% CI) | P(x) | d(x) | LE, years (95% CI) | P(x) | d(x) | LE, years 95% CI) | P(x) | d(x) | LE, years (95% CI) | P(x) | d(x) | LE , years (95% CI) |
| 0–1 | 1,815 | 1,366 | 1.54 (1.49–1.6) | 16,426 | 6,905 | 3.91 (3.86–3.97) | 1,429,166 | 33,080 | 13.18 (13.16–13.19) | 149,434 | 142 | 13.14 (13.12–13.16) | 6,078 | 2 | 11.71 (11.66–11.77) |
| 1–2 | 520 | 225 | 2.16 (2.07–2.26) | 7,434 | 1,100 | 4.89 (4.82–4.96) | 1,107,541 | 11,810 | 12.48 (12.47–12.49) | 204,373 | 446 | 12.15 (12.14–12.17) | 8,425 | 18 | 10.72 (10.67–10.77) |
| 2–3 | 417 | 174 | 2.08 (1.98–2.18) | 5,666 | 866 | 4.59 (4.52–4.66) | 917,654 | 8,857 | 11.61 (11.6–11.62) | 240,622 | 885 | 11.18 (11.16–11.2) | 10,702 | 50 | 9.74 (9.69–9.79) |
| 3–4 | 367 | 194 | 1.92 (1.82–2.01) | 4,670 | 772 | 4.27 (4.2–4.33) | 806,362 | 8,453 | 10.72 (10.7–10.73) | 266,749 | 1,551 | 10.22 (10.2–10.23) | 13,212 | 131 | 8.78 (8.73–8.83) |
| 4–5 | 376 | 184 | 1.94 (1.85–2.03) | 4,269 | 788 | 3.94 (3.88–4.0) | 752,826 | 8,977 | 9.82 (9.81–9.83) | 291,878 | 2,525 | 9.27 (9.26–9.29) | 15,734 | 263 | 7.87 (7.82–7.91) |
| 5–6 | 406 | 203 | 1.87 (1.79–1.95) | 4,388 | 910 | 3.64 (3.59–3.7) | 686,235 | 10,321 | 8.93 (8.92–8.95) | 297,657 | 3,858 | 8.35 (8.33–8.37) | 17,687 | 440 | 6.99 (6.94–7.04) |
| 6–7 | 428 | 228 | 1.78 (1.71–1.86) | 4,660 | 1,077 | 3.37 (3.32–3.42) | 620,330 | 11,653 | 8.06 (8.05–8.07) | 289,853 | 5,550 | 7.45 (7.44–7.47) | 18,446 | 716 | 6.15 (6.11–6.2) |
| 7–8 | 567 | 312 | 1.72 (1.65–1.78) | 5,438 | 1,411 | 3.12 (3.08–3.16) | 578,600 | 14,686 | 7.21 (7.19–7.22) | 282,704 | 8,305 | 6.59 (6.57–6.6) | 18,858 | 1,118 | 5.38 (5.33–5.42) |
| 8–9 | 689 | 413 | 1.64 (1.59–1.69) | 6,657 | 1,928 | 2.9 (2.87–2.94) | 524,971 | 19,652 | 6.38 (6.37–6.39) | 257,944 | 11,445 | 5.77 (5.75–5.79) | 18,138 | 1,573 | 4.67 (4.63–4.72) |
| 9–10 | 648 | 374 | 1.62 (1.56–1.67) | 7,284 | 2,169 | 2.72 (2.69–2.75) | 460,179 | 24,892 | 5.6 (5.59–5.61) | 222,199 | 15,094 | 5.01 (4.99–5.02) | 15,990 | 1,959 | 4.05 (4.01–4.1) |
| 10–11 | 880 | 564 | 1.52 (1.48–1.56) | 8,926 | 3,053 | 2.49 (2.47–2.52) | 401,897 | 30,595 | 4.89 (4.87–4.9) | 187,142 | 18,589 | 4.33 (4.31–4.34) | 13,860 | 2,302 | 3.52 (3.47–3.56) |
| 11–12 | 865 | 543 | 1.48 (1.44–1.52) | 9,147 | 3,297 | 2.32 (2.29–2.34) | 321,975 | 33,904 | 4.23 (4.22–4.25) | 140,708 | 19,548 | 3.73 (3.71–3.74) | 10,404 | 2,241 | 3.06 (3.01–3.11) |
| 12–13 | 1,043 | 726 | 1.38 (1.35–1.41) | 10,363 | 4,154 | 2.11 (2.09–2.13) | 250,004 | 36,720 | 3.65 (3.64–3.66) | 100,923 | 19,421 | 3.21 (3.19–3.23) | 7,417 | 2,162 | 2.68 (2.63–2.74) |
| 13–14 | 1,259 | 921 | 1.32 (1.29–1.34) | 10,949 | 4,837 | 1.92 (1.91–1.94) | 181,725 | 36,586 | 3.15 (3.13–3.16) | 65,150 | 16,697 | 2.78 (2.76–2.8) | 4,683 | 1,612 | 2.43 (2.36–2.49) |

| | | | | | | | | | | | | | | | |
|---|---|---|---|---|---|---|---|---|---|---|---|---|---|---|---|
| 14–15 | 1,249 | 945 | 1.26 (1.24–1.28) | 9,786 | 5,073 | 1.73 (1.71–1.75) | 117,064 | 31,356 | 2.74 (2.72–2.76) | 36,828 | 12,087 | 2.45 (2.43–2.48) | 2,440 | 984 | 2.23 (2.14–2.31) |
| 15–16 | 1,167 | 964 | 1.19 (1.17–1.21) | 7,276 | 4,264 | 1.59 (1.57–1.61) | 64,569 | 22,076 | 2.43 (2.41–2.45) | 17,496 | 6,917 | 2.22 (2.19–2.25) | 1,168 | 561 | 2.1 (1.98–2.22) |
| 16–17 | 761 | 644 | 1.15 (1.13–1.18) | 4,190 | 2,659 | 1.5 (1.47–1.52) | 29,650 | 12,217 | 2.23 (2.2–2.25) | 6,966 | 3,114 | 2.07 (2.03–2.11) | 445 | 218 | 2.11 (1.93–2.29) |
| 17+ | 820 | 737 | 1.11 (1.09–1.14) | 3,606 | 2,537 | 1.42 (1.39–1.45) | 17,340 | 8,153 | 2.13 (2.09–2.16) | 3,584 | 1,816 | 1.97 (1.91–2.04) | 291 | 135 | 2.16 (1.89–2.42) |

BCS, body condition score; CI, confidence interval; d(x), deceased study population in the age interval (x, x + 1); LE, life expectancy; P(x), total population (survivors plus deceased) at the mid-point of the age interval (x, x + 1).

**Supplementary Table 12. Life expectancies of cats by age interval and body condition score**

| Age interval, years | BCS 1 | | | BCS 2 | | | BCS 3 | | | BCS 4 | | | BCS 5 | | |
|---|---|---|---|---|---|---|---|---|---|---|---|---|---|---|---|
| | P(x) | d(x) | LE, years (95% CI) | P(x) | d(x) | LE, years (95% CI) | P(x) | d(x) | LE, years (95% CI) | P(x) | d(x) | LE, years (95% CI) | P(x) | d(x) | LE, years (95% CI) |
| 0–1 | 1,564 | 1,359 | 1.04 (1.01–1.06) | 6,567 | 4,281 | 1.75 (1.72–1.79) | 270,914 | 10,904 | 12.18 (12.14–12.21) | 41,530 | 93 | 13.67 (13.62–13.71) | 2,545 | 2 | 12.56 (12.45–12.66) |
| 1–2 | 498 | 391 | 1.33 (1.28–1.37) | 2,467 | 1,263 | 2.26 (2.21–2.31) | 168,231 | 4,995 | 11.67 (11.63–11.7) | 55,711 | 416 | 12.7 (12.65–12.74) | 3,551 | 22 | 11.57 (11.46–11.67) |
| 2–3 | 376 | 272 | 1.4 (1.34–1.45) | 1,974 | 890 | 2.47 (2.41–2.53) | 120,441 | 3,327 | 11.0 (10.97–11.04) | 57,688 | 716 | 11.79 (11.75–11.84) | 4,319 | 49 | 10.63 (10.53–10.73) |
| 3–4 | 328 | 240 | 1.41 (1.35–1.47) | 1,828 | 760 | 2.61 (2.55–2.68) | 96,248 | 2,728 | 10.3 (10.26–10.33) | 58,420 | 877 | 10.93 (10.89–10.98) | 5,243 | 97 | 9.75 (9.65–9.85) |
| 4–5 | 305 | 208 | 1.46 (1.4–1.53) | 2,036 | 794 | 2.72 (2.66–2.79) | 83,082 | 2,522 | 9.58 (9.55–9.61) | 58,184 | 998 | 10.09 (10.05–10.13) | 6,283 | 159 | 8.92 (8.83–9.01) |
| 5–6 | 381 | 274 | 1.46 (1.4–1.52) | 2,568 | 973 | 2.8 (2.74–2.86) | 72,411 | 2,618 | 8.86 (8.83–8.89) | 55,969 | 1,196 | 9.26 (9.21–9.3) | 7,035 | 213 | 8.14 (8.05–8.23) |
| 6–7 | 424 | 271 | 1.54 (1.48–1.6) | 2,888 | 1,010 | 2.88 (2.82–2.93) | 62,742 | 2,433 | 8.17 (8.13–8.2) | 51,689 | 1,314 | 8.44 (8.4–8.49) | 7,128 | 248 | 7.37 (7.29–7.46) |
| 7–8 | 515 | 344 | 1.51 (1.46–1.56) | 3,691 | 1,284 | 2.88 (2.83–2.93) | 58,328 | 2,654 | 7.47 (7.44–7.5) | 48,573 | 1,503 | 7.65 (7.61–7.69) | 7,427 | 356 | 6.62 (6.53–6.7) |
| 8–9 | 748 | 503 | 1.53 (1.48–1.57) | 4,796 | 1,617 | 2.89 (2.84–2.93) | 55,319 | 3,151 | 6.79 (6.76–6.83) | 44,235 | 1,866 | 6.87 (6.83–6.92) | 7,148 | 423 | 5.92 (5.84–6.0) |
| 9–10 | 724 | 431 | 1.57 (1.52–1.61) | 5,302 | 1,688 | 2.86 (2.82–2.89) | 50,300 | 3,279 | 6.16 (6.13–6.19) | 37,894 | 1,999 | 6.15 (6.11–6.19) | 6,394 | 487 | 5.25 (5.17–5.33) |
| 10–11 | 1,236 | 848 | 1.47 (1.44–1.51) | 8,116 | 2,873 | 2.75 (2.72–2.78) | 49,970 | 4,389 | 5.55 (5.52–5.57) | 34,144 | 2,420 | 5.46 (5.41–5.5) | 5,779 | 580 | 4.62 (4.54–4.7) |
| 11–12 | 1,099 | 716 | 1.49 (1.45–1.52) | 8,086 | 2,640 | 2.71 (2.68–2.74) | 43,708 | 4,140 | 5.01 (4.98–5.04) | 26,954 | 2,416 | 4.82 (4.78–4.86) | 4,614 | 586 | 4.06 (3.98–4.14) |
| 12–13 | 1,569 | 1,077 | 1.44 (1.41–1.47) | 10,227 | 3,532 | 2.58 (2.55–2.6) | 41,385 | 5,044 | 4.46 (4.43–4.49) | 22,266 | 2,813 | 4.22 (4.18–4.27) | 3,951 | 679 | 3.54 (3.46–3.62) |
| 13–14 | 1,772 | 1,181 | 1.42 (1.4–1.45) | 11,639 | 4,123 | 2.44 (2.42–2.47) | 36,686 | 5,597 | 3.97 (3.94–4.0) | 17,208 | 2,708 | 3.73 (3.68–3.77) | 2,896 | 653 | 3.11 (3.02–3.2) |

| | | | | | | | | | | | | | | | |
|---|---|---|---|---|---|---|---|---|---|---|---|---|---|---|---|
| 14–15 | 2,115 | 1,526 | 1.34 (1.32–1.37) | 12,771 | 4,910 | 2.28 (2.26–2.3) | 31,178 | 5,785 | 3.54 (3.51–3.57) | 12,491 | 2,598 | 3.28 (3.23–3.33) | 2,025 | 556 | 2.78 (2.68–2.87) |
| 15–16 | 2,468 | 1,846 | 1.3 (1.28–1.32) | 12,909 | 5,410 | 2.13 (2.11–2.15) | 25,169 | 5,808 | 3.17 (3.13–3.2) | 8,487 | 2,173 | 2.92 (2.87–2.98) | 1,237 | 419 | 2.5 (2.39–2.62) |
| 16–17 | 2,367 | 1,813 | 1.25 (1.24–1.27) | 11,292 | 5,022 | 1.99 (1.97–2.01) | 17,644 | 4,966 | 2.86 (2.83–2.9) | 5,053 | 1,626 | 2.63 (2.57–2.7) | 756 | 283 | 2.32 (2.18–2.46) |
| 17+ | 6,413 | 5,414 | 1.18 (1.17–1.2) | 22,028 | 11,936 | 1.85 (1.82–1.87) | 22,539 | 8,549 | 2.64 (2.59–2.68) | 4,809 | 1,961 | 2.45 (2.37–2.54) | 627 | 291 | 2.15 (1.97–2.34) |

BCS, body condition score; CI, confidence interval; d(x), deceased study population in the age interval (x, x + 1); LE, life expectancy; P(x), total population (survivors plus deceased) at the mid-point of the age interval (x, x + 1).

**Supplementary Table 13. Life expectancies at birth of dogs and cats by survey year and body condition score**

| Year of survey | BCS 1 | BCS 2 | BCS 3 | BCS 4 | BCS 5 |
|---|---|---|---|---|---|
| **Dogs LE at birth, years (95% CI)** | | | | | |
| 2013 | 1.73 (1.61–1.85) | 4.35 (4.22–4.47) | 13.73 (13.68–13.78) | 13.01 (12.95–13.06) | 11.47 (11.3–11.63) |
| 2014 | 1.57 (1.44–1.7) | 3.86 (3.74–3.98) | 13.04 (13.0–13.08) | 12.8 (12.76–12.85) | 11.08 (10.94–11.22) |
| 2015 | 1.32 (1.21–1.44) | 3.67 (3.53–3.8) | 13.08 (13.04–13.11) | 12.85 (12.81–12.9) | 11.2 (11.08–11.33) |
| 2016 | 1.48 (1.31–1.64) | 3.78 (3.63–3.93) | 13.09 (13.06–13.12) | 13.13 (13.09–13.17) | 11.79 (11.62–11.96) |
| 2017 | 1.49 (1.32–1.67) | 3.81 (3.65–3.96) | 13.17 (13.14–13.2) | 13.2 (13.16–13.24) | 12.02 (11.89–12.16) |
| 2018 | 1.48 (1.31–1.64) | 3.91 (3.74–4.07) | 13.21 (13.18–13.24) | 13.35 (13.31–13.4) | 12.24 (12.11–12.38) |
| 2019 | 1.52 (1.35–1.69) | 3.58 (3.43–3.73) | 13.15 (13.12–13.18) | 13.46 (13.42–13.51) | 12.57 (12.42–12.72) |
| **Cats LE at birth, years (95% CI)** | | | | | |
| 2013 | 1.07 (1.01–1.12) | 2.11 (2.01–2.21) | 12.88 (12.76–13.01) | 13.23 (13.08–13.38) | 12.12 (11.85–12.39) |
| 2014 | 0.99 (0.95–1.04) | 1.79 (1.71–1.87) | 11.87 (11.77–11.97) | 12.97 (12.84–13.09) | 11.58 (11.31–11.85) |
| 2015 | 1.06 (1.0–1.13) | 1.69 (1.61–1.77) | 12.01 (11.91–12.1) | 13.33 (13.21–13.45) | 12.15 (11.88–12.42) |
| 2016 | 1.08 (1.0–1.16) | 1.7 (1.61–1.79) | 11.96 (11.87–12.05) | 13.83 (13.71–13.95) | 12.52 (12.25–12.78) |
| 2017 | 1.01 (0.95–1.08) | 1.7 (1.61–1.79) | 12.09 (12.01–12.18) | 13.77 (13.66–13.88) | 13.04 (12.77–13.32) |
| 2018 | 0.98 (0.92–1.04) | 1.66 (1.58–1.75) | 12.47 (12.38–12.56) | 14.11 (14.0–14.22) | 13.23 (12.96–13.5) |

| | | | | | |
|---|---|---|---|---|---|
| 2019 | 1.06 (0.98–1.15) | 1.51 (1.44–1.59) | 12.19 (12.1–12.27) | 14.19 (14.08–14.3) | 13.41 (13.1–13.72) |

BCS, body condition score; CI, confidence interval; LE, life expectancy.
